# An Updated Taxonomy of *Talaromyces* (Trichocomaceae, Eurotiales): New Series and Species

**DOI:** 10.3390/jof12070485

**Published:** 2026-07-01

**Authors:** Lu-Yao Peng, He Song, Yi-Fan Wang, Wen-Ying Zhuang, Xin-Cun Wang

**Affiliations:** 1 State Key Laboratory of Microbial Diversity and Innovative Utilization, Institute of Microbiology, Chinese Academy of Sciences, Beijing 100101, China; pengluyao327@163.com (L.-Y.P.); zhuangwy@im.ac.cn (W.-Y.Z.); 2 University of Chinese Academy of Sciences, Beijing 100049, China; 3 College of Modern Agriculture and Ecological Environment, Heilongjiang University, Harbin 150080, China; 13104631380@163.com; 4 School of Biotechnology, Jiangnan University, Wuxi 214122, China; yfwang.bai@foxmail.com; 5 Department of Nutrition and Health, China Agricultural University, Beijing 100083, China

**Keywords:** ascomycota, new species, phylogeny, taxonomic system

## Abstract

Species of *Talaromyces* affect human societies in many different ways. Infrageneric classifications of the genus at series level had been established in only two sections, *Subinflati* and *Trachyspermi*. In this study, phylogenies of *Talaromyces* were reconstructed section by section based on separate or concatenated multi-locus datasets: beta-tubulin (*BenA*), calmodulin (*CaM*) and RNA polymerase II second-largest subunit (*RPB2*). Fifty series belonging to nine sections were accordingly recognized, i.e., one in sections *Brunneospori* and *Tenues*, two in sections *Bacillispori* and *Helici*, three in sect. *Subinflati*, four in sect. *Purpurei*, five in sections *Islandici* and *Trachyspermi*, and 27 in the speciose section *Talaromyces*. Among them, forty series were newly established. Three new species were determined phylogenetically and morphologically, i.e., *T. fujianensis* sp. nov. in sect. *Islandici*, *T. heilongjiangensis* sp. nov. in sect. *Talaromyces* and *T. tapisciae* sp. nov. in sect. *Subinflati*. Additionally, three new Chinese records were noted: *T. angelicae*, *T. gautengensis* and *T. rogersiae*. The findings of new species and new records reveal the high diversity of the genus in China. The updated taxonomy of *Talaromyces* at series level will facilitate a more accurate species identification by means of phylogenetic analysis at a smaller scale, and benefit future studies involving this group of fungi.

## 1. Introduction

Species of *Talaromyces* C.R. Benj. are cosmopolitan and ubiquitous, and their multiple functions as both friend and foe have been well documented. New compounds isolated from the marine-derived fungus *T. minnesotensis* Guevara-Suarez et al. showed synergistic antibacterial activity against *Staphylococcus aureus* [1], and new cytotoxic γ-lactam alkaloids from the mangrove-derived fungus *T. hainanensis* K. Hong & Ling Liu were reported to have potential for developing antihepatocellular carcinoma agents [2]. *Talaromyces albobiverticillius* (H.M. Hsieh et al.) Samson et al. demonstrated the production of plant growth regulating compounds like Indole Acetic Acid (IAA) and proficient solubilization of crucial nutrients [3]. *Talaromyces cystophila* Y.X. Mo & H.Y. Wu was deemed as a potential biocontrol agent with nematophagous and nematicidal activity against corn cyst nematode [4]. *Talaromyces benedictus* D.S. Paiva isolated from limestone surfaces of a Portuguese church might play a considerable role in the deterioration of cultural heritage [5]. *Talaromyces marneffei* (Segretain et al.) Samson et al., a life-threatening dimorphic fungus, causes systemic mycosis in Southeast Asia [6]. Nearly 17,300 cases of *T. marneffei* infection are diagnosed annually, and the mortality rate is extremely high at 1/3 [7].

This genus was established in 1955, and infrageneric classifications were subsequently proposed. Four sections were divided in 1972, primarily based on the structure of the conidial state: *Emersonii*, *Purpurea*, *Talaromyces* and *Thermophila* [8]. Section *Trachyspermi* was further established in 1996 based on the ubiquinone systems [9]. Seven sections were recognized in 2014 based on an ITS, *BenA* and *RPB2* multigene phylogeny, which has been widely adopted: *Bacillispori*, *Helici*, *Islandici*, *Purpurei*, *Subinflati*, *Talaromyces* and *Trachyspermi* [10]. Sections *Tenues* and *Brunneospori* were recently added [11,12]. At series level, five series were established based on colonial color and growth rate: *Flavi*, *Lutei* and *Trachyspermi* in sect. *Talaromyces sensu* Stolk & Samson, *Purpurei* in sect. *Purpurea*, and *Thermophili* in sect. *Thermophila* [13]. Eight series were recently proposed based on phylogenetic analyses inferred from multi-locus datasets (ITS, *BenA*, *CaM* and *RPB2*): *Palmarum*, *Resedani* and *Subinflati* in sect. *Subinflati* and *Diversi*, *Erythromelles*, *Miniolutei*, *Resinarum* and *Trachyspermi* (emended) in sect. *Trachyspermi* [14]. The infrageneric classification at series level of *Talaromyces* based on multi-locus phylogeny provided a more refined system for phylogenetic comparison and species determination.

A total of 88 species of the genus were accepted in 2014 [10], and the number increased to 171 in 2020 [15], to 203 by 2023 [16], and to 236 by April 2025 [12]. More recently, seven species were further added: five from China (*T. elephas* X.C. Wang et al., *T. pseudorugulosus* Q.M. Wang et al., *T. sinensis* X.C. Wang et al., *T. taiwanensis* K.W. Cheng & H.A. Ariyaw. and *T. xishuangbannaensis* X.C. Wang et al.), *T. ignescens* Van Vuuren et al. from South Africa and *T. tianshanicus* X.C. Wang et al. from Uzbekistan. The accelerated increase in species number of the genus further requires a more refined taxonomy at series level.

This study is aimed at (i) reconstructing a multi-locus phylogeny of *Talaromyces* section by section and providing an updated taxonomy at series level and (ii) exploring species diversity of the genus in China through examinations of the recent collections molecularly and morphologically.

## 2. Materials and Methods

### 2.1. Fungal Materials

Cultures were isolated from soil samples collected from several Chinese provinces (Fujian, Hebei, Heilongjiang, Xinjiang and Yunnan) or as culture contaminant in the lab of Beijing, China, during 2015 to 2025. Dried cultures were preserved in the Herbarium Mycologicum Academiae Sinicae (HMAS, Beijing, China), and the living ex-type strains were deposited in the China General Microbiological Culture Collection Center (CGMCC, Beijing, China).

### 2.2. Morphological Observations

Morphological characteristics were observed and recorded according to standardized methods [17]. Four standard growth media were adopted: Czapek yeast autolysate agar (CYA, yeast extract Oxoid, Hampshire, UK), malt extract agar (MEA, Amresco, Solon, OH, USA), yeast extract agar (YES) and potato dextrose agar (PDA). The methods for colonial inoculation, incubation, macroscopic and microscopic examinations and digital capture followed our previous studies [18,19].

### 2.3. DNA Extraction, PCR Amplification and Sequencing

DNA was extracted from living cultures grown on PDA for 7 days using the Plant Genomic DNA Kit (DP305, TIANGEN Biotech, Beijing, China). Polymerase chain reaction (PCR) amplifications of four gene partitions, i.e., internal transcribed spacer (ITS), beta-tubulin (*BenA*), calmodulin (*CaM*) and RNA polymerase II second-largest subunit (*RPB2*), were conducted with routine methods [17]. The products were sequenced on an ABI 3730 DNA Sequencer (Applied Biosystems, Foster, CA, USA).

### 2.4. Phylogenetic Analyses

The newly generated forward and reverse sequences in this research were assembled by Seqman v. 7.1.0 (DNASTAR Inc., Madison, WI, USA). The assembled sequences were deposited at GenBank with the given accessions in bold (Table 1, Table 2, Table 3, Table 4, Table 5 and Table 6). The additional sequences used for phylogenetic analyses are also listed. Sequences, either from each of the three single-gene datasets (*BenA*, *CaM* and *RPB2*) or from the concatenated ones, were aligned using MAFFT v. 7.221 [20]. Subsequently, they were manually edited and concatenated in BioEdit v. 7.1.10 [21] and MEGA v. 11.0.13 [22]. Maximum likelihood (ML) analyses were performed using the IQ-TREE v. 3.0.1 (https://doi.org/10.32942/X2P62N, accessed on 17 March 2026) with the default Auto substitution model and 1000 bootstrap (BP) iteration settings. Bayesian inference (BI) analyses were conducted with MrBayes v. 3.2.7 [23]. Modeltest v. 3.7 [24] was adopted to determine appropriate nucleotide substitution models and parameters. Four MCMC chains (three heated ones and one cold chain) were run for at least 1 million generations, and posterior probability (PP) values were calculated based on the remaining 75% of trees after the burn-in phase. The consensus trees were viewed using FigTree v. 1.4.4 (http://tree.bio.ed.ac.uk/software/figtree (accessed on 28 December 2023)).

## 3. Results

To reconstruct the phylogenies of sections in *Talaromyces*, the single-gene datasets (*BenA*, *CaM* and *RPB2*) and the concatenated ones were compiled and analyzed. The detailed characteristics of the datasets are summarized in Table 7.

**Figure 1:**
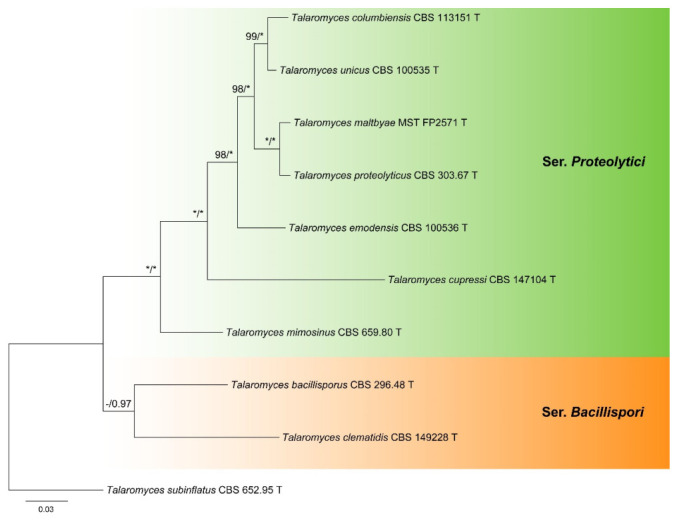
Maximum likelihood phylogeny of *Talaromyces* sect. *Bacillispori* inferred from the combined *BenA*, *CaM* and *RPB2* dataset. Bootstrap values ≥ 70% (**left**) or posterior probability values ≥ 0.95 (**right**) are indicated at nodes. Asterisk denotes 100% bootstrap or 1.00 posterior probability.

As shown in Figure 1, two clades were clearly recognized with significant statistic supports in the phylogenetic tree of *Talaromyces* sect. *Bacillispori* inferred from the multi-gene dataset, which should represent two series in this section. The phylogenies based on individual genes also supported their separation (Figures S1–S3).

**Figure 2:**
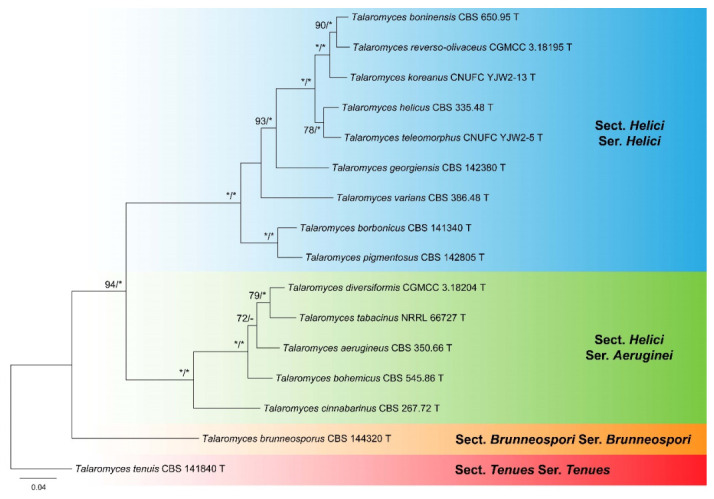
Maximum likelihood phylogeny of sections *Brunneospori*, *Helici* and *Tenues* in *Talaromyces* inferred from the combined *BenA*, *CaM* and *RPB2* dataset. Bootstrap values ≥ 70% (**left**) or posterior probability values ≥ 0.95 (**right**) are indicated at nodes. Asterisk denotes 100% bootstrap or 1.00 posterior probability.

In Figure 2, the sections *Helici*, *Brunneospori* and *Tenues* are well distinguished, and two clades are noticeable with strong statistic supports, representing different series in sect. *Helici*. The phylogenies based on individual genes are provided in Figures S4–S6.

**Figure 3:**
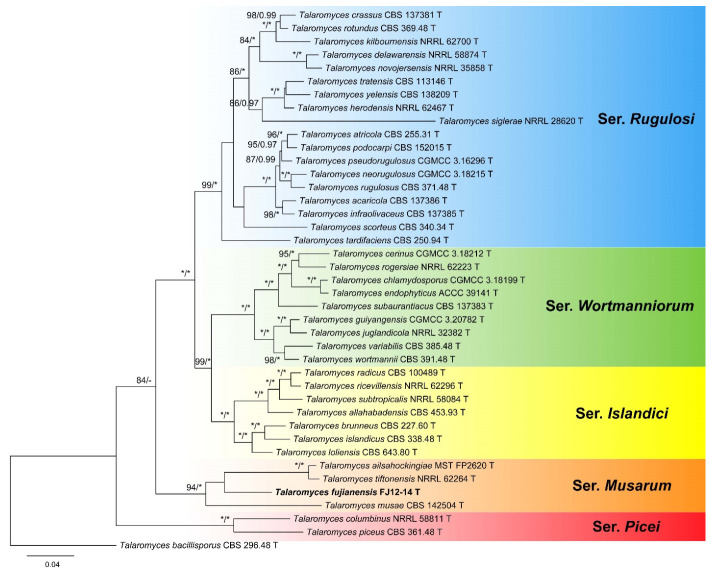
Maximum likelihood phylogeny of *Talaromyces* sect. *Islandici* inferred from the combined *BenA*, *CaM* and *RPB2* dataset. Bootstrap values ≥ 70% (**left**) or posterior probability values ≥ 0.95 (**right**) are indicated at nodes. Asterisk denotes 100% bootstrap or 1.00 posterior probability.

Five clades were revealed in *Talaromyces* sect. *Islandici*, and all of them were strongly supported based on the combined dataset (Figure 3). They should be distinguished at series level. The strain FJ12-14 formed an independent lineage of one clade and thus represents a new species. The phylogenies of sect. *Islandici* based on individual genes are given in Figures S7–S9.

**Figure 4:**
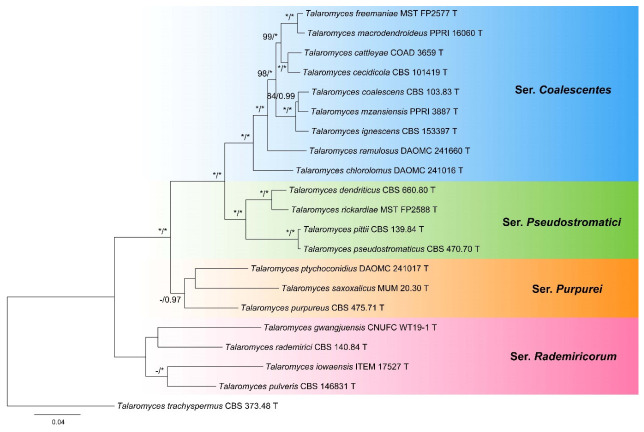
Maximum likelihood phylogeny of *Talaromyces* sect. *Purpurei* inferred from the combined *BenA*, *CaM* and *RPB2* dataset. Bootstrap values ≥ 70% (**left**) or posterior probability values ≥ 0.95 (**right**) are indicated at nodes. Asterisk denotes 100% bootstrap or 1.00 posterior probability.

Four clades were divided in *Talaromyces* sect. *Purpurei* and represented different series, although two of them did not receive high supports (Figure 4). The phylogenies based on individual genes were presented in Figures S10–S12.

**Figure 5:**
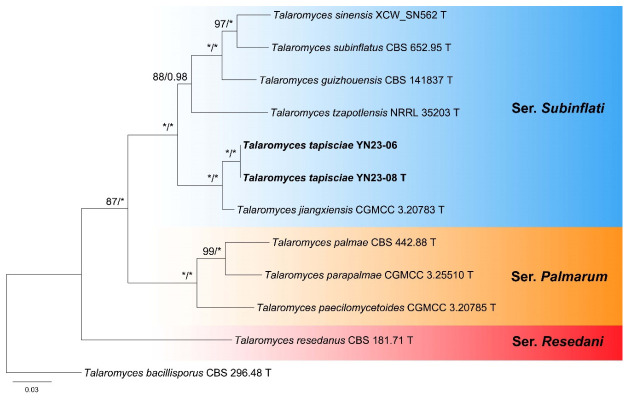
Maximum likelihood phylogeny of *Talaromyces* sect. *Subinflati* inferred from the combined *BenA*, *CaM* and *RPB2* dataset. Bootstrap values ≥ 70% (**left**) or posterior probability values ≥ 0.95 (**right**) are indicated at nodes. Asterisk denotes 100% bootstrap or 1.00 posterior probability.

In the phylogeny of *Talaromyces* sect. *Subinflati* (Figure 5), three clades were supported. A proposed new species was represented by strains YN23-06 and YN23-08, which were clustered with *T. jiangxiensis*, also distributed in China. The phylogenies based on individual genes are shown in Figures S13–S15.

**Figure 6:**
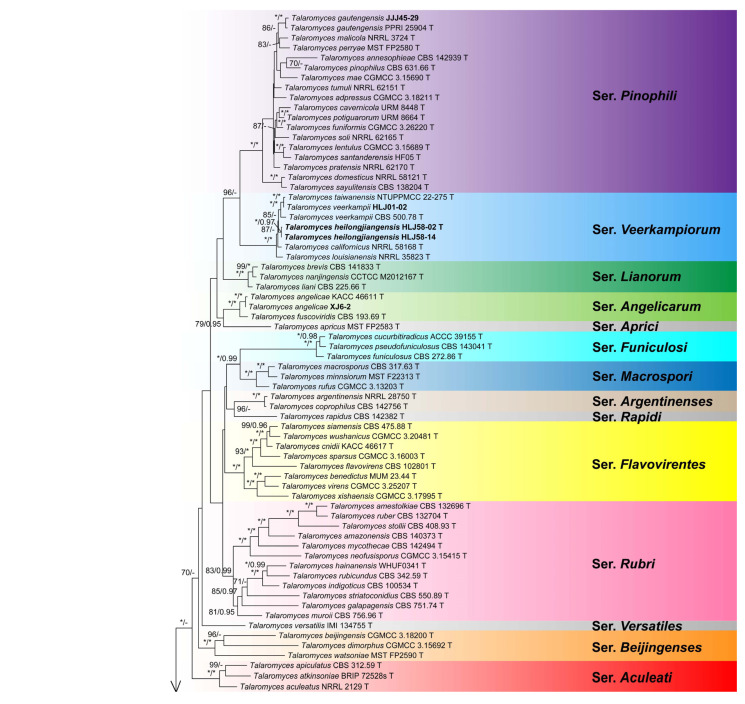

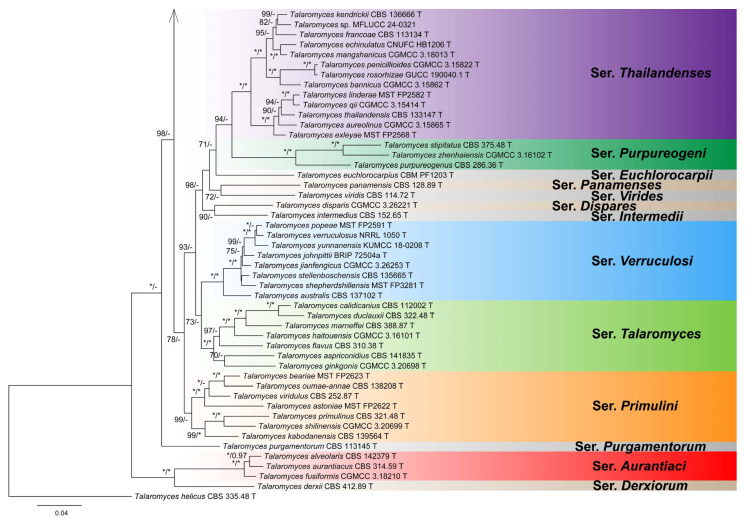
Maximum likelihood phylogeny of *Talaromyces* sect. *Talaromyces* inferred from the combined *BenA*, *CaM* and *RPB2* dataset. Bootstrap values ≥ 70% (**left**) or posterior probability values ≥ 0.95 (**right**) are indicated at nodes. Asterisk denotes 100% bootstrap or 1.00 posterior probability.

More than 110 species were included in the phylogeny of *Talaromyces* sect. *Talaromyces* and 27 clades or independent lineages were clearly revealed (Figure 6). The strains HLJ58-02 and HLJ58-14 represented an undescribed species in the clade consisting of *T. veerkampii* and its allies. The phylogenies based on individual genes can be seen in Figures S16–S18.

## 4. Taxonomy

### 4.1. New Series

***Talaromyces*** C.R. Benj., Mycologia 47(5): 681, 1955.

**Section *Bacillispori*** N. Yilmaz, Frisvad & Samson, Stud. Mycol. 78: 191, 2014.

Series ***Bacillispori*** X.C. Wang & W.Y. Zhuang, **ser. nov.**

Fungal Names: FN573766

Etymology: Named after the type species of the series, *Talaromyces bacillisporus*.

Type species: *Talaromyces bacillisporus* (Swift) C.R. Benj., Mycologia 47(5): 684, 1955.

≡ *Penicillium bacillisporum* Swift, Bull. Torrey Bot. Club 59: 221, 1932.

Accepted species: *Talaromyces bacillisporus*, *T. clematidis*.

Notes: Series *Bacillispori* was monophyletic in the combined and single *CaM* phylogenies (Figure 1 and Figure S2) but not in *BenA* or *RPB2* tree (Figures S1 and S3). The two members are both isolated from plant materials.

Series ***Proteolytici*** X.C. Wang & W.Y. Zhuang, **ser. nov.**

Fungal Names: FN573767

Etymology: Named after the type species of the series, *Talaromyces proteolyticus*.

Type species: *Talaromyces proteolyticus* (Kamyschko) Samson, N. Yilmaz & Frisvad, Stud. Mycol. 70: 176, 2011.

≡ *Penicillium proteolyticum* Kamyschko, Notul. Syst. Sect. Cryptog. Inst. Bot. Acad. Sci. U.S.S.R. 14: 228, 1961.

Accepted species: *Talaromyces columbiensis*, *T. cupressi*, *T. emodensis*, *T. maltbyae*, *T. mimosinus*, *T. proteolyticus*, *T. unicus*.

Notes: Series *Proteolytici* represents the main body of the section and contains seven species. Most of them are isolated from soil.

**Section *Brunneospori*** Visagie, Houbraken & Hubka, Stud. Mycol. 112: 130, 2025.

Series ***Brunneospori*** X.C. Wang & W.Y. Zhuang, **ser. nov.**

Fungal Names: FN573768

Etymology: Named after the type species of the series, *Talaromyces brunneosporus*.

Type species: *Talaromyces brunneosporus* Rodr.-Andr., Cano & Stchigel, IMA Fungus 10(20): 19, 2019.

Accepted species: *Talaromyces brunneosporus*.

Notes: The series was established to accommodate the type species of the section, and it is sister to ser. *Tenues* of sect. *Tenues* (Figure 2 and Figures S4–S6).

**Section *Helici*** N. Yilmaz, Frisvad & Samson, Stud. Mycol. 78: 189, 2014.

Series ***Aeruginei*** X.C. Wang & W.Y. Zhuang, **ser. nov.**

Fungal Names: FN573769

Etymology: Named after the type species of the series, *Talaromyces aerugineus*.

Type species: *Talaromyces aerugineus* (Samson) N. Yilmaz, Frisvad & Samson, Stud. Mycol. 78: 210, 2014.

≡ *Paecilomyces aerugineus* Samson, Stud. Mycol. 6: 20, 1974.

Accepted species: *Talaromyces aerugineus*, *T. bohemicus*, *T. cinnabarinus*, *T. diversiformis*, *T. tabacinus*.

Notes: Five members are included in ser. *Aeruginei* and *T. cinnabarinus* located as the basal lineage in the combined phylogeny and trees based on *BenA* or *RPB2* individually (Figure 2, Figures S4 and S6). *Talaromyces ryukyuensis* (S. Ueda & Udagawa) Arx, having only ITS sequence without any of the protein-coding genes, could also be placed in the series [12].

Series ***Helici*** X.C. Wang & W.Y. Zhuang, **ser. nov.**

Fungal Names: FN573770

Etymology: Named after the type species of the series, *Talaromyces helicus*.

Type species: *Talaromyces helicus* (Raper & Fennell) C.R. Benj., Mycologia 47(5): 684, 1955.

≡ *Penicillium helicum* Raper & Fennell, Mycologia 40(5): 515, 1948.

Accepted species: *Talaromyces boninensis*, *T. borbonicus*, *T. georgiensis*, *T. helicus*, *T. koreanus*, *T. pigmentosus*, *T. reverso-olivaceus*, *T. teleomorphus*, *T. varians*.

Notes: Series *Helici* can further be divided into three parts: one containing *T. borbonicus* and *T. pigmentosus* as the basal subclade, another consisting of *T. georgiensis* and *T. varians*, and the rest species forming the last part.

**Section *Islandici*** (Pitt) N. Yilmaz, Frisvad & Samson, Stud. Mycol. 78: 192, 2014.

Series ***Islandici*** X.C. Wang & W.Y. Zhuang, **ser. nov.**

Fungal Names: FN573771

Etymology: Named after the type species of the series, *Talaromyces islandicus*.

Type species: *Talaromyces islandicus* (Sopp) Samson, N. Yilmaz, Frisvad & Seifert, Stud. Mycol. 70: 176, 2011.

≡ *Penicillium islandicum* Sopp, Skr. VidenskSelsk. Christiania, Kl. I, Math.-Natur. (no. 11): 161, 1912.

Accepted species: *Talaromyces allahabadensis*, *T. brunneus*, *T. islandicus*, *T. loliensis*, *T. radicus*, *T. ricevillensis*, *T. subtropicalis*.

Notes: Series *Islandici* was monophyletic in the combined phylogeny and single-gene *CaM* or *RPB2* tree (Figure 3, Figures S8 and S9), which was not monophyletic based on *BenA* sequence analysis (Figure S7). It was sister to ser. *Wortmanniorum* (Figure 3, Figures S7 and S9).

Series ***Musarum*** X.C. Wang & W.Y. Zhuang, **ser. nov.**

Fungal Names: FN573772

Etymology: Named after the type species of the series, *Talaromyces musae*.

Type species: *Talaromyces musae* Houbraken, Kraak & M. Meijer, Persoonia 39: 341, 2017.

Accepted species: *Talaromyces ailsahockingiae*, *T. fujianensis*, *T. musae*, *T. tiftonensis*.

Notes: Series *Musarum* was a monophyly in all analyses and contained four members, including the newly introduced taxon *T. fujianensis* (Figure 3 and Figures S7–S9).

Series ***Picei*** X.C. Wang & W.Y. Zhuang, **ser. nov.**

Fungal Names: FN573773

Etymology: Named after the type species of the series, *Talaromyces piceus*.

Type species: *Talaromyces piceus* (Raper & Fennell) Samson, N. Yilmaz, Houbraken, Spierenb., Seifert, Peterson, Varga & Frisvad, Stud. Mycol. 70: 176, 2011.

≡ *Penicillium piceum* Raper & Fennell, Mycologia 40(5): 533, 1948.

Accepted species: *Talaromyces columbinus*, *T. piceus*.

Notes: Series *Picei* appeared to be the basal clade of the section and sister to ser. *Musarum* (Figure 3 and Figure S8). Only two species are currently recognized.

Series ***Rugulosi*** X.C. Wang & W.Y. Zhuang, **ser. nov.**

Fungal Names: FN573774

Etymology: Named after the type species of the series, *Talaromyces rugulosus*.

Type species: *Talaromyces rugulosus* (Thom) Samson, N. Yilmaz, Frisvad & Seifert, Stud. Mycol. 70: 177, 2011.

≡ *Penicillium rugulosum* Thom, Bull. U.S. Department of Agriculture 118: 60, 1910.

Accepted species: *Talaromyces acaricola*, *T. atricola*, *T. crassus*, *T. delawarensis*, *T. herodensis*, *T. infraolivaceus*, *T. kilbournensis*, *T. neorugulosus*, *T. novojersensis*, *T. podocarpi*, *T. pseudorugulosus*, *T. rotundus*, *T. rugulosus*, *T. scorteus*, *T. siglerae*, *T. tardifaciens*, *T. tratensis*, *T. yelensis*.

Notes: Series *Rugulosi* is most speciose in the section and with 18 taxa currently known. It was monophyletic in the multi-locus, *CaM* and *RBP2* trees but polyphyletic in the BenA analysis (Figure 3 and Figures S7–S9).

Series ***Wortmanniorum*** X.C. Wang & W.Y. Zhuang, **ser. nov.**

Fungal Names: FN573775

Etymology: Named after the type species of the series, *Talaromyces wortmannii*.

Type species: *Talaromyces wortmannii* (Klöcker) C.R. Benj., Mycologia 47(5): 683, 1955.

≡ *Penicillium wortmannii* Klöcker, C. r. Trav. Laboratoire d. Carlsberg 6: 100, 1906.

Accepted species: *Talaromyces cerinus*, *T. chlamydosporus*, *T. endophyticus*, *T. guiyangensis*, *T. juglandicola*, *T. rogersiae*, *T. subaurantiacus*, *T. variabilis*, *T. wortmannii*.

Notes: Series *Wortmanniorum* was sister to ser. *Islandici* (Figure 3, Figures S7 and S9), and with nine species currently known.

**Section *Purpurei*** Stolk & Samson, Stud. Mycol. 2: 56, 1972.

Series ***Coalescentes*** X.C. Wang & W.Y. Zhuang, **ser. nov.**

Fungal Names: FN573776

Etymology: Named after the type species of the series, *Talaromyces coalescens*.

Type species: *Talaromyces coalescens* (Quintan.) Samson, N. Yilmaz & Frisvad, Stud. Mycol. 70: 175, 2011.

≡ *Penicillium coalescens* Quintan., Mycopathologia 84(2-3): 115, 1984.

Accepted species: *Talaromyces cattleyae*, *T. cecidicola*, *T. chlorolomus*, *T. coalescens*, *T. freemaniae*, *T. ignescens*, *T. macrodendroideus*, *T. mzansiensis*, *T. ramulosus*.

Notes: Series *Coalescentes* was monophyletic and sister to ser. *Pseudostromatici* with strong supports in all the analyses (Figure 4 and Figures S10–S12). The series has a worldwide distribution.

Series ***Pseudostromatici*** X.C. Wang & W.Y. Zhuang, **ser. nov.**

Fungal Names: FN573777

Etymology: Named after the type species of the series, *Talaromyces pseudostromaticus*.

Type species: *Talaromyces pseudostromaticus* (Hodges, G.M. Warner & Rogerson) Samson, N. Yilmaz, Frisvad & Seifert, Stud. Mycol. 70: 176, 2011.

≡ *Penicillium pseudostromaticum* Hodges, G.M. Warner & Rogerson, Mycologia 62(6): 1106, 1971.

Accepted species: *Talaromyces dendriticus*, *T. pittii*, *T. pseudostromaticus*, *T. rickardiae*.

Notes: Series *Pseudostromatici* was monophyletic and sister to ser. *Coalescentes* receiving strong statistic supports in all the analyses (Figure 4 and Figures S10–S12). It has a worldwide distribution.

Series ***Purpurei*** Pitt, The Genus *Penicillium* and its teleomorph states *Eupenicillium* and *Talaromyces* (London): 512, 1979.

Type species: *Talaromyces purpureus* (E. Müll. & Pacha-Aue) Stolk & Samson, Stud. Mycol. 2: 57, 1972.

≡ *Arachniotus purpureus* E. Müll. & Pacha-Aue, Nova Hedwigia 15: 552, 1968.

Accepted species: *Talaromyces ptychoconidius*, *T. purpureus*, *T. saxoxalicus*.

Notes: Series *Purpurei* was established to place *T. purpureus* because of its dark red mycelia produced on MEA at 25 °C [13]. This series was monophyletic in the combined phylogeny except for *RPB2* analysis (Figure 4 and Figure S12). Two new members were added.

Series ***Rademiricorum*** X.C. Wang & W.Y. Zhuang, **ser. nov.**

Fungal Names: FN573778

Etymology: Named after the type species of the series, *Talaromyces rademirici*.

Type species: *Talaromyces rademirici* (Quintan.) Samson, N. Yilmaz & Frisvad, Stud. Mycol. 70: 177, 2011.

≡ *Penicillium rademirici* Quintan., Mycopathologia 91(2): 72, 1985.

Accepted species: *Talaromyces gwangjuensis*, *T. iowaensis*, *T. pulveris*, *T. rademirici*.

Notes: Series *Rademiricorum* was the basal clade in the section and included four members. It was monophyletic in the combined and single *BenA* analyses but did not receive high statistic supports (Figure 4 and Figure S10) and appeared as paraphyletic in the *RPB2* phylogeny (Figure S12).

**Section *Subinflati*** N. Yilmaz, Frisvad & Samson, Stud. Mycol. 78: 192, 2014.

Series ***Palmarum*** X.C. Wang & W.Y. Zhuang, J. Fungi 11(7, no. 508): 10, 2025.

Type species: *Talaromyces palmae* (Samson, Stolk & Frisvad) Samson, N. Yilmaz, Frisvad & Seifert, Stud. Mycol. 70: 176, 2011.

≡ *Penicillium palmae* Samson, Stolk & Frisvad, Stud. Mycol. 31: 135, 1989.

Accepted species: *Talaromyces paecilomycetoides*, *T. palmae*, *T. parapalmae*.

Notes: The concept of the series was shown in the previous study [14].

Series ***Resedani*** X.C. Wang & W.Y. Zhuang, J. Fungi 11(7, no. 508): 10, 2025.

Type species: *Talaromyces resedanus* (McLennan & Ducker) A.J. Chen, Houbraken & Samson, MycoKeys 68: 96, 2020.

≡ *Penicillium resedanum* McLennan & Ducker, Aust. J. Bot. 2(3): 360, 1954.

Accepted species: *Talaromyces resedanus*.

Notes: The concept of the series was stated in the previous study [14].

Series ***Subinflati*** X.C. Wang & W.Y. Zhuang, J. Fungi 11(7, no. 508): 10, 2025.

Type species: *Talaromyces subinflatus* Yaguchi & Udagawa, Trans. Mycol. Soc. Japan 34(2): 249, 1993.

Accepted species: *Talaromyces guizhouensis*, *T. jiangxiensis*, *T. sinensis*, *T. subinflatus*, *T. tapisciae*, *T. tzapotlensis*.

Notes: The concept of the series was stated in the previous study [14]. *Talaromyces tapisciae* from China was newly added.

**Section *Talaromyces*** C.R. Benj., Mycologia 47(5): 681, 1955.

Series ***Aculeati*** X.C. Wang, **ser. nov.**

Fungal Names: FN573779

Etymology: Named after the type species of the series, *Talaromyces aculeatus*.

Type species: *Talaromyces aculeatus* (Raper & Fennell) Samson, N. Yilmaz, Frisvad & Seifert, Stud. Mycol. 70: 174, 2011.

≡ *Penicillium aculeatum* Raper & Fennell, Mycologia 40(5): 535, 1948.

Accepted species: *Talaromyces aculeatus*, *T. apiculatus*, *T. atkinsoniae*.

Notes: Series *Aculeati* was monophyletic in both combined and single-gene analyses (Figure 6 and Figures S16–S18). It is distributed worldwide.

Series ***Angelicarum*** X.C. Wang, **ser. nov.**

Fungal Names: FN573780

Etymology: Named after the type species of the series, *Talaromyces angelicae*.

Type species: *Talaromyces angelicae* S.H. Yu, T.J. An & H.K. Sang, J. Microbiol. 51(5): 707, 2013.

Accepted species: *Talaromyces angelicae*, *T. fuscoviridis*.

Notes: Series *Angelicarum* was monophyletic in all the analyses. It was sister to ser. *Aprici* in the combined and *CaM* inferences (Figure 6 and Figures S16–S18).

Series ***Aprici*** X.C. Wang, **ser. nov.**

Fungal Names: FN573781

Etymology: Named after the type species of the series, *Talaromyces apricus*.

Type species: *Talaromyces apricus* Y.P. Tan, Minns & E. Lacey, Index of Australian Fungi 34: 7, 2024.

Accepted species: *Talaromyces apricus*.

Notes: Series *Aprici* was sister to ser. *Angelicarum* in the combined and *CaM* phylogenies, which was not supported by the *BenA* and *RPB2* analyses (Figure 6 and Figures S16–S18). In the *BenA* tree, *T. apricus* grouped with the members of ser. *Rubri* (Figure S16).

Series ***Argentinenses*** X.C. Wang, **ser. nov.**

Fungal Names: FN573782

Etymology: Named after the type species of the series, *Talaromyces argentinensis*.

Type species: *Talaromyces argentinensis* Jurjević & S.W. Peterson, Fungal Biol. 123(10): 751, 2019.

Accepted species: *Talaromyces argentinensis*, *T. coprophilus*,

Notes: Series *Argentinenses* was monophyletic, with strong supports in all analyses. Its close relationship with ser. *Rapidi* was revealed by the multi-locus phylogeny but not by single-gene analyses (Figure 6 and Figures S16–S18).

Series ***Aurantiaci*** X.C. Wang, **ser. nov.**

Fungal Names: FN573783

Etymology: Named after the type species of the series, *Talaromyces aurantiacus*.

Type species: *Talaromyces aurantiacus* (J.H. Mill., Giddens & A.A. Foster) Samson, N. Yilmaz & Frisvad, Stud. Mycol. 70: 175, 2011.

≡ *Penicillium aurantiacum* J.H. Mill., Giddens & A.A. Foster, Mycologia 49(6): 797, 1958.

Accepted species: *Talaromyces alveolaris*, *T. aurantiacus*, *T. fusiformis*.

Notes: Series *Aurantiaci* is sister to ser. *Derxiorum* with strong supports (Figure 6 and Figures S16–S18). Both were the basal clades in the combined phylogeny.

Series ***Beijingenses*** X.C. Wang, **ser. nov.**

Fungal Names: FN573784

Etymology: Named after the type species of the series, *Talaromyces beijingensis*.

Type species: *Talaromyces beijingensis* A.J. Chen, Frisvad & Samson, Stud. Mycol. 84: 125, 2016.

Accepted species: *Talaromyces beijingensis*, *T. dimorphus*, *T. watsoniae*.

Notes: Series *Beijingenses* was monophyletic in the combined and single-gene analyses (Figure 6 and Figures S16–S18). Two of the three known species are from China and the other one from Australia.

Series ***Derxiorum*** X.C. Wang, **ser. nov.**

Fungal Names: FN573785

Etymology: Named after the type species of the series, *Talaromyces derxii*.

Type species: *Talaromyces derxii* Takada & Udagawa, Mycotaxon 31(2): 418, 1988.

Accepted species: *Talaromyces derxii*.

Notes: Series *Derxiorum* is sister to ser. *Aurantiaci* with strong supports (Figure 6 and Figures S16–S18). They were the basal clades in the combined phylogeny. *Talaromyces derxii* was the first *Talaromyces* species to be heterothallic and produced green ascomata and spiny ellipsoidal ascospores [10].

Series ***Dispares*** X.C. Wang, **ser. nov.**

Fungal Names: FN573786

Etymology: Named after the type species of the series, *Talaromyces disparis*.

Type species: *Talaromyces disparis* Y.M. Ruan & L. Wang, PeerJ 12(e18253): 8, 2024.

Accepted species: *Talaromyces disparis*.

Notes: Ser. *Dispares* was an independent lineage and phylogenetically close to ser. *Intermedii* in the combined phylogeny (Figure 6). But the relationship was not supported by all the single-gene analyses (Figures S16–S18).

Series ***Euchlorocarpii*** X.C. Wang, **ser. nov.**

Fungal Names: FN573787

Etymology: Named after the type species of the series, *Talaromyces euchlorocarpius*.

Type species: *Talaromyces euchlorocarpius* Yaguchi, Someya & Udagawa, Mycoscience 40(2): 133, 1999.

Accepted species: *Talaromyces euchlorocarpius*.

Notes: Ser. *Euchlorocarpii* was an independent lineage and close to ser. *Purpureogeni* and ser. *Thailandenses* in the combined phylogeny (Figure 6).

Series ***Flavovirentes*** X.C. Wang, **ser. nov.**

Fungal Names: FN573788

Etymology: Named after the type species of the series, *Talaromyces flavovirens*.

Type species: *Talaromyces flavovirens* (Durieu & Mont.) Visagie, Llimona & Seifert, Mycotaxon 122: 404, 2013.

≡ *Lasioderma flavovirens* Durieu & Mont., Annls Sci. Nat., Bot., sér. 3, 4(no. 96): 364, 1845.

Accepted species: *Talaromyces benedictus*, *T. cnidii*, *T. flavovirens*, *T. siamensis*, *T. sparsus*, *T. virens*, *T. wushanicus*, *T. xishaensis*.

Notes: Series *Flavovirentes* was monophyletic in the combined phylogeny as well as single *CaM* analysis, which did not agree with the *BenA* and *RPB2* trees (Figure 6 and Figures S16–S18). Among the eight known species, six of them are from Asia, including four from China.

Series ***Funiculosi*** X.C. Wang, **ser. nov.**

Fungal Names: FN573789

Etymology: Named after the type species of the series, *Talaromyces funiculosus*.

Type species: *Talaromyces funiculosus* (Thom) Samson, N. Yilmaz, Frisvad & Seifert, Stud. Mycol. 70: 176, 2011.

≡ *Penicillium funiculosum* Thom, Bull. U.S. Department of Agriculture, Bureau Animal Industry 118: 69, 1910.

Accepted species: *Talaromyces cucurbitiradicus*, *T. funiculosus*, *T. pseudofuniculosus*.

Notes: Series *Funiculosi* was monophyletic in all the analyses and sister to ser. *Macrospori* in the combined phylogeny (Figure 6 and Figures S16–S18).

Series ***Intermedii*** X.C. Wang, **ser. nov.**

Fungal Names: FN573790

Etymology: Named after the type species of the series, *Talaromyces intermedius*.

Type species: *Talaromyces intermedius* (Apinis) Stolk & Samson, Stud. Mycol. 2: 21, 1972.

≡ *Arachniotus intermedius* Apinis, Mycol. Pap. 96: 45, 1964.

Accepted species: *Talaromyces intermedius*.

Notes: Ser. *Intermedii* represented an independent lineage (Figure 6). Its relationship with ser. *Dispares* was discussed above.

Series ***Lianorum*** X.C. Wang, **ser. nov.**

Fungal Names: FN573791

Etymology: Named after the type species of the series, *Talaromyces liani*.

Type species: *Talaromyces liani* (Kamyschko) N. Yilmaz, Frisvad & Samson, Stud. Mycol. 78: 266, 2014.

≡ *Penicillium liani* Kamyschko, Notul. syst. Sect. cryptog. Inst. bot. Acad. Sci. U.S.S.R. 15: 86, 1962.

Accepted species: *Talaromyces brevis*, *T. liani*, *T. nanjingensis*.

Notes: Series *Lianorum* was monophyletic in all the analyses (Figure 6 and Figures S16–S18). They all occur in China.

Series ***Macrospori*** X.C. Wang, **ser. nov.**

Fungal Names: FN573792

Etymology: Named after the type species of the series, *Talaromyces macrosporus*.

Type species: *Talaromyces macrosporus* (Stolk & Samson) Frisvad, Samson & Stolk, Antonie van Leeuwenhoek 57: 186, 1990.

≡ *Talaromyces flavus* var. *macrosporus* Stolk & Samson, Stud. Mycol. 2: 15, 1972.

Accepted species: *Talaromyces macrosporus*, *T. minnsiorum*, *T. rufus*.

Notes: Series *Macrospori* appeared to be monophyletic, with strong supports (Figure 6 and Figures S16–S18) and sister to ser. *Funiculosi* in the combined tree (Figure 6).

Series ***Panamenses*** X.C. Wang, **ser. nov.**

Fungal Names: FN573793

Etymology: Named after the type species of the series, *Talaromyces panamensis*.

Type species: *Talaromyces panamensis* (Samson, Stolk & Frisvad) Samson, N. Yilmaz, Frisvad & Seifert, Stud. Mycol. 70: 176, 2011.

≡ *Penicillium panamense* Samson, Stolk & Frisvad, Stud. Mycol. 31: 136, 1989.

Accepted species: *Talaromyces panamensis*.

Notes: Ser. *Panamenses* was an independent lineage and phylogenetically close to ser. *Virides* in the combined phylogeny (Figure 6). But this relationship was not supported by all the single-gene analyses (Figures S16–S18).

Series ***Pinophili*** X.C. Wang, **ser. nov.**

Fungal Names: FN573794

Etymology: Named after the type species of the series, *Talaromyces pinophilus*.

Type species: *Talaromyces pinophilus* (Hedgc.) Samson, N. Yilmaz, Frisvad & Seifert, Stud. Mycol. 70: 176, 2011.

≡ *Penicillium pinophilum* Hedgc., Bull. U.S. Department of Agriculture, Bureau Animal Industry 118: 75, 1910.

Accepted species: *Talaromyces adpressus*, *T. annesophieae*, *T. cavernicola*, *T. domesticus*, *T. funiformis*, *T. gautengensis*, *T. lentulus*, *T. mae*, *T. malicola*, *T. perryae*, *T. pinophilus*, *T. potiguarorum*, *T. pratensis*, *T. santanderensis*, *T. sayulitensis*, *T. soli*, *T. tumuli*.

Notes: Series *Pinophili* was well-defined in the combined and single *CaM* and *RPB2* phylogenies, which was not supported by the *BenA* analysis (Figure 6 and Figures S16–S18). It is most speciose in the section and contains 17 known species.

Series ***Primulini*** X.C. Wang, **ser. nov.**

Fungal Names: FN573795

Etymology: Named after the type species of the series, *Talaromyces primulinus*.

Type species: *Talaromyces primulinus* (Pitt) Samson, N. Yilmaz & Frisvad, Stud. Mycol. 70: 176, 2011.

≡ *Penicillium primulinum* Pitt, The Genus *Penicillium* and its teleomorph states *Eupenicillium* and *Talaromyces*: 455, 1979.

Accepted species: *Talaromyces astoniae*, *T. beariae*, *T. kabodanensis*, *T. oumae-annae*, *T. primulinus*, *T. shilinensis*, *T. viridulus*.

Notes: Series *Primulini* was monophyletic in the combined and single *RPB2* phylogenies, which was different from the *BenA* and *CaM* analyses (Figure 6 and Figures S16–S18). Among the known species, three are from Oceania and two from Asia.

Series ***Purgamentorum*** X.C. Wang, **ser. nov.**

Fungal Names: FN573796

Etymology: Named after the type species of the series, *Talaromyces purgamentorum*.

Type species: *Talaromyces purgamentorum* N. Yilmaz, López-Quint., Vasco-Pal. & Houbraken, Mycol. Progr. 15: 1054, 2016.

Accepted species: *Talaromyces purgamentorum*.

Notes: Series *Purgamentorum* is shown as an independent lineage in the combined phylogeny and single *BenA* and *RPB2* trees (Figure 6, Figures S16 and S18). It was mixed with species of ser. *Primulini* in the *CaM* analysis (Figure S17).

Series ***Purpureogeni*** X.C. Wang, **ser. nov.**

Fungal Names: FN573797

Etymology: Named after the type species of the series, *Talaromyces purpureogenus*.

Type species: *Talaromyces purpureogenus* (Stoll) Samson, N. Yilmaz, Houbraken, Spierenb., Seifert, Peterson, Varga & Frisvad, Stud. Mycol. 70: 177, 2011.

≡ *Penicillium purpureogenum* Stoll, Beitr. Morph. Biol. Char. Penicillium: 32, 1904.

Accepted species: *Talaromyces purpureogenus*, *T. stipitatus*, *T. zhenhaiensis*.

Notes: Series *Purpureogeni* was monophyletic in the combined and single-gene phylogenies. It was sister to ser. *Thailandenses* in the combined and single *BenA* and *RPB2* analyses, but this series became a basal clade in the *CaM* phylogeny (Figure 6 and Figures S16–S18).

Series ***Rapidi*** X.C. Wang, **ser. nov.**

Fungal Names: FN573798

Etymology: Named after the type species of the series, *Talaromyces rapidus*.

Type species: *Talaromyces rapidus* Guevara-Suarez, Dania García & Gené, Mycoses 60(10): 658, 2017.

Accepted species: *Talaromyces rapidus*.

Notes: Series *Rapidi* was sister to ser. *Argentinenses* in the combined phylogeny (Figure 6), which was not supported by the single-gene analyses (Figures S16–S18).

Series ***Rubri*** X.C. Wang, **ser. nov.**

Fungal Names: FN573799

Etymology: Named after the type species of the series, *Talaromyces ruber*.

Type species: *Talaromyces ruber* (Stoll) N. Yilmaz, Houbraken, Frisvad & Samson, Persoonia 29: 48, 2012.

≡ *Penicillium rubrum* Stoll, Beitr. Morph. Biol. Char. Penicillium: 35, 1904.

Accepted species: *Talaromyces amazonensis*, *T. amestolkiae*, *T. galapagensis*, *T. hainanensis*, *T. indigoticus*, *T. muroii*, *T. mycothecae*, *T. neofusisporus*, *T. ruber*, *T. rubicundus*, *T. stollii*, *T. striatoconidius*.

Notes: Series *Rubri* was supported by the combined phylogeny (Figure 6). In the *BenA* tree, this series was highly supported (MLBP = 98), somehow, *T. apricus* of ser. *Aprici* joined in (Figure S16). The monophyly of the series was not supported by the *CaM* and *RPB2* analyses (Figures S17 and S18).

Series *Talaromyces* C.R. Benj., Mycologia 47(5): 681, 1955.

Type species: *Talaromyces flavus* (Klöcker) Stolk & Samson, Stud. Mycol. 2: 10, 1972.

≡ *Gymnoascus flavus* Klöcker, Hedwigia 41: 80, 1902.

Accepted species: *Talaromyces aspriconidius*, *T. calidicanius*, *T. duclauxii*, *T. flavus*, *T. ginkgonis*, *T. haitouensis*, *T. marneffei*.

Notes: Series *Talaromyces* is mainly distributed in East Asia, e.g., *T. aspriconidius*, *T. calidicanius*, *T. ginkgonis* and *T. haitouensis* from China and *T. marneffei* originally described from Vietnam. The monophyly of the series was not supported by single *CaM* analysis (Figure S17). This series does not correspond to the concept of series *Flavi sensu* Pitt [13], to which the following species belong: *T. flavus*, *T. helicus* (in section *Helici*), *T. stipitatus*, *T. panasenkoi* (=*T. ucrainicus* in section *Trachyspermi*), and *T. striatus* (≡ *Pseudohamigera striata*).

Series ***Thailandenses*** X.C. Wang, **ser. nov.**

Fungal Names: FN573800

Etymology: Named after the type species of the series, *Talaromyces thailandensis*.

Type species: *Talaromyces thailandensis* Manoch, Dethoup & N. Yilmaz, Mycoscience 54(5): 339, 2013.

Accepted species: *Talaromyces aureolinus*, *T. bannicus*, *T. echinulatus*, *T. exleyae*, *T. francoae*, *T. kendrickii*, *T. linderae*, *T. mangshanicus*, *T. penicillioides*, *T. qii*, *T. thailandensis*.

Notes: Ser. *Thailandenses* was monophyletic in both combined phylogeny and single-gene analyses with strong supports. Its sister relationship with ser. *Purpureogeni* was well-supported except for the *CaM* analysis (Figure 6 and Figures S16–S18). *Talaromyces rosorhizae nom. inval.* belonged to this series, as well as the recently introduced species *T. doitungensis* Thakshila et al. from Thailand (*Talaromyces* sp. MFLUCC 24-0321) [25].

Series ***Veerkampiorum*** X.C. Wang, **ser. nov.**

Fungal Names: FN573801

Etymology: Named after the type species of the series, *Talaromyces veerkampii*.

Type species: *Talaromyces veerkampii* Visagie, N. Yilmaz & Samson, Mycoscience 56: 497, 2015.

Accepted species: *Talaromyces californicus*, *T. heilongjiangensis*, *T. louisianensis*, *T. veerkampii*.

Notes: Series *Veerkampiorum* was monophyletic in all the analyses with strong supports (Figure 6 and Figures S16–S18). A new species *T. heilongjiangensis* was introduced in the series, and *T. taiwanensis* was treated as a later synonym of *T. veerkampii* in view of the very limited sequence divergence.

Series ***Verruculosi*** X.C. Wang, **ser. nov.**

Fungal Names: FN573802

Etymology: Named after the type species of the series, *Talaromyces verruculosus*.

Type species: *Talaromyces verruculosus* (Peyronel) Samson, N. Yilmaz, Frisvad & Seifert, Stud. Mycol. 70: 177, 2011.

≡ *Penicillium verruculosum* Peyronel, I germi astmosferici dei fungi con micelio, Diss.: 22, 1913.

Accepted species: *Talaromyces australis*, *T. jianfengicus*, *T. johnpittii*, *T. popeae*, *T. shepherdshillensis*, *T. stellenboschensis*, *T. verruculosus*, *T. yunnanensis*.

Notes: Series *Verruculosi* was monophyletic in the combined and single-gene phylogenies, but the sister relationship between *T. australis* and the other members of the series was poorly supported in the *RPB2* analysis (Figure S18). This series was sister to ser. *Talaromyces* in the combined phylogeny (Figure 6), which was not supported by the single-gene phylogenies (Figures S16–S18).

Series ***Versatiles*** X.C. Wang, **ser. nov.**

Fungal Names: FN573803

Etymology: Named after the type species of the series, *Talaromyces versatilis*.

Type species: *Talaromyces versatilis* Bridge & Buddie, Index Fungorum 26: 1, 2013.

Accepted species: *Talaromyces versatilis*.

Notes: Series *Versatiles* appeared as an independent lineage in the combined phylogeny (Figure 6). But it clustered with ser. *Angelicarum* in the *BenA* tree, grouped with ser. *Beijingenses* in *CaM* tree, and was close to some species of ser. *Rubri* in *RPB2* tree (Figures S16–S18).

Series ***Virides*** X.C. Wang, **ser. nov.**

Fungal Names: FN573804

Etymology: Named after the type species of the series, *Talaromyces viridis*.

Type species: *Talaromyces viridis* (Stolk & G.F. Orr) Arx, Persoonia 13(3): 282, 1987.

≡ *Sagenoma viride* Stolk & G.F. Orr, Mycologia 66(4): 677, 1974.

Accepted species: *Talaromyces viridis*.

Notes: Ser. *Virides* represented as an independent lineage (Figure 6). Its relationship with ser. *Panamenses* has been discussed previously.

**Section *Tenues*** B.D. Sun, A.J. Chen, Houbraken & Samson, MycoKeys 68: 82, 2020.

Series ***Tenues*** X.C. Wang & W.Y. Zhuang, **ser. nov.**

Fungal Names: FN573805

Etymology: Named after the type species of the series, *Talaromyces tenuis*.

Type species: *Talaromyces tenuis* B.D. Sun, A.J. Chen, Houbraken & Samson, MycoKeys 68: 86, 2020.

Accepted species: *Talaromyces tenuis*.

Notes: The series was established to accommodate only the type species of the section, and it was sister to ser. *Brunneospori* of sect. *Brunneospori* (Figure 2 and Figures S4–S6).

**Section *Trachyspermi*** Yaguchi & Udagawa, Mycoscience 37(1): 57, 1996.

Series ***Diversi*** X.C. Wang & W.Y. Zhuang, J. Fungi 11(7, no. 508): 10, 2025.

Type species: *Talaromyces diversus* (Raper & Fennell) Samson, N. Yilmaz & Frisvad, Stud. Mycol. 70: 175, 2011.

≡ *Penicillium diversum* Raper & Fennell, Mycologia 40(5): 539, 1948.

Accepted species: *Talaromyces albisclerotius*, *T. clemensii*, *T. cystophila*, *T. diversus*, *T. peaticola*, *T. tianshanicus*.

Notes: The concept of the series has been discussed previously [14].

Series ***Erythromelles*** X.C. Wang & W.Y. Zhuang, J. Fungi 11(7, no. 508): 10, 2025.

Type species: *Talaromyces erythromellis* (A.D. Hocking) Samson, N. Yilmaz, Frisvad & Seifert, Stud. Mycol. 70: 175, 2011.

≡ *Penicillium erythromellis* A.D. Hocking, The genus *Penicillium* and its teleomorph states *Eupenicillium* and *Talaromyces*: 459, 1979.

Accepted species: *Talaromyces aerius*, *T. albobiverticillius*, *T. amyrossmaniae*, *T. austrocalifornicus*, *T. catalonicus*, *T. convolutus*, *T. elephas*, *T. erythromellis*, *T. heiheensis*, *T. pernambucoensis*, *T. rubidus*, *T. rubrifaciens*, *T. solicola*.

Notes: The concept of the series has been stated in the previous study [14].

Series ***Miniolutei*** X.C. Wang & W.Y. Zhuang, J. Fungi 11(7, no. 508): 11, 2025.

Type species: *Talaromyces minioluteus* (Dierckx) Samson, N. Yilmaz, Frisvad & Seifert, Stud. Mycol. 70: 176, 2011.

≡ *Penicillium minioluteum* Dierckx, Ann. Soc. Sci. Bruxelles 25: 87, 1901.

Accepted species: *Talaromyces africanus*, *T. calidominioluteus*, *T. chongqingensis*, *T. gaditanus*, *T. germanicus*, *T. minioluteus*, *T. minnesotensis*, *T. samsonii*, *T. udagawae*, *T. xishuangbannaensis*.

Notes: The series has been discussed in the previous study [14].

Series ***Resinarum*** X.C. Wang & W.Y. Zhuang, J. Fungi 11(7, no. 508): 11, 2025.

Type species: *Talaromyces resinae* (Z.T. Qi & H.Z. Kong) Houbraken & X.C. Wang, Stud. Mycol. 95: 91, 2020.

≡ *Penicillium resinae* Z.T. Qi & H.Z. Kong, Acta Mycol. Sin. 1(2): 103, 1982.

Accepted species: *Talaromyces brasiliensis*, *T. longistipes*, *T. phuphaphetensis*, *T. resinae*, *T. satunensis*, *T. subericola*.

Notes: This series has been discussed in the previous study [14].

Series ***Trachyspermi*** Pitt sensu Peng et al., J. Fungi 11(7, no. 508): 11, 2025.

Type species: *Talaromyces trachyspermus* (Shear) Stolk & Samson, Stud. Mycol. 2: 32, 1973.

≡ *Arachniotus trachyspermus* Shear, Science 16: 138, 1902.

Accepted species: *Talaromyces albidus*, *T. affinitatimellis*, *T. assiutensis*, *T. atroroseus*, *T. basipetosporus*, *T. ellipsoideus*, *T. guatemalensis*, *T. hallidayae*, *T. mellisjaponici*, *T. phialiformis*, *T. speluncarum*, *T. systylus*, *T. trachyspermus*, *T. ucrainicus*.

Notes: This series has been discussed in the previous study [14].

### 4.2. New Species

**Figure 7:**
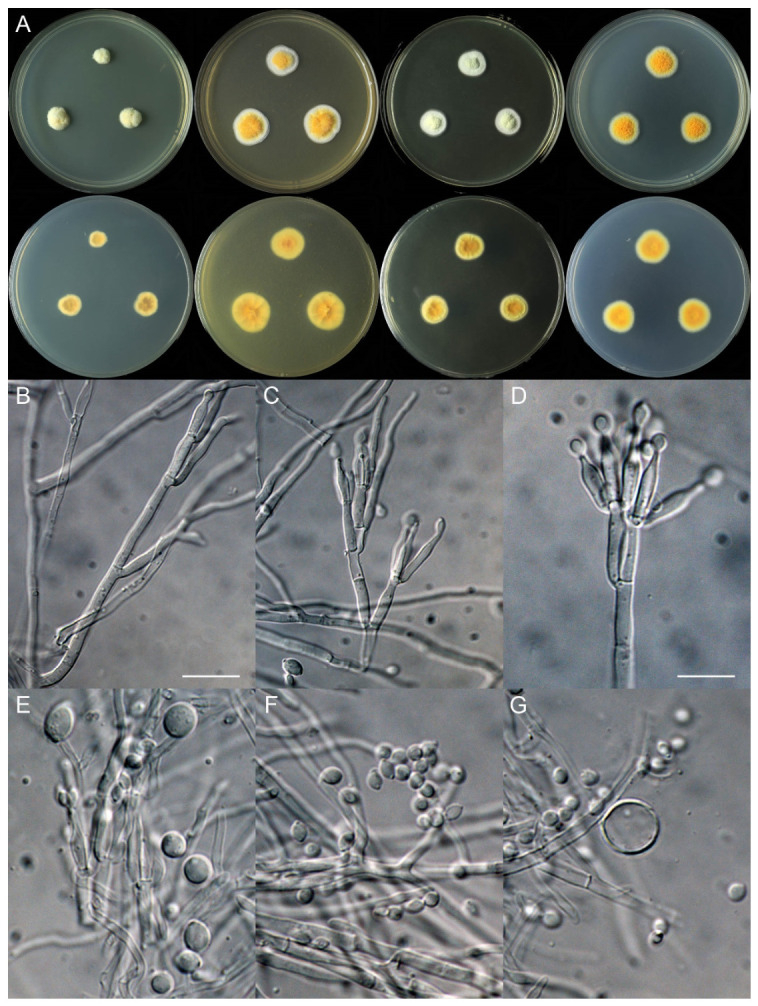
*Talaromyces fujianensis* (FJ12-14). (**A**) Colonies: top row left to right, obverse CYA, MEA, YES, and PDA; bottom row left to right, reverse CYA, MEA, YES, and PDA; (**B**–**D**) conidiophores; (**E**) macroconidia; (**F**) microconidia; (**G**) chlamydospore. Bars: (**B**) = 15 µm, also for (**C**); (**D**) = 10 µm, also for (**E**–**G**).

***Talaromyces fujianensis*** X.C. Wang, L.Y. Peng & W.Y. Zhuang, **sp. nov.** Figure 7

Fungal Names: FN573764

Etymology: The specific epithet refers to the type locality of the fungus.

In *Talaromyces* sect. *Islandici* ser. *Musarum*

Typification: CHINA. Fujian Province, Zhangzhou City, Nanjing County, Huboliao National Nature Reserve, Jinshan Town, E’xiandong (goose fairy cave), 24°47′25″ N 117°20′6″ E, soil under rock, 15 July 2025, Xin-Cun Wang, culture, Lu-Yao Peng, FJ12-14 (holotype HMAS 354375, preserved in a metabolically inactive state; ex-type strain CGMCC 3.29867).

DNA barcodes: ITS PZ326302, *BenA* PZ321402, *CaM* PZ321406, *RPB2* PZ321412.

Colony diam., 7 days, 25 °C (unless stated otherwise): CYA 9–12 mm; CYA 37 °C no growth; CYA 5 °C no growth; MEA 17–19 mm; YES 15–16 mm; PDA 16–17 mm.

Colony characteristics: On CYA 25 °C, 7 days: Colonies irregular, protuberant, deep; margins narrow, irregular; mycelia buff; texture velutinous and sticky; sporulation absent; soluble pigments absent; exudates absent; reverse yellow brown to dull brown.

On MEA 25 °C, 7 days: Colonies irregular, protuberant at centers; margins moderately wide, entire or fimbriate; mycelia white, yellow at centers; texture velutinous, floccose at centers, sticky; sporulation absent; soluble pigments absent; exudates absent; reverse white to yellow.

On YES 25 °C, 7 days: Colonies irregular, protuberant at centers; margins narrow, entire; mycelia pale, light yellow at centers; texture velutinous and sticky; sporulation sparse; conidia en masse light grey; soluble pigments absent; exudates absent; reverse yellow brown, white at margins; strong odor.

On PDA 25 °C, 7 days: Colonies nearly irregular, protuberant at centers; margins moderately wide, entire to fimbriate; mycelia white, yellow at centers; texture velutinous, floccose at centers, sticky; sporulation absent; soluble pigments absent; exudates absent; reverse white to yellow to orange.

Micromorphology: Conidiophores biverticillate, terverticillate or more branched; stipes smooth-walled, 30–70 × 2.0–3.5 μm; rami 2, 15–33.5 × 2.5–3.0 μm; metulae 2–3, 10–32.5 × 2.5–3.5 μm; phialides acerose or ampulliform, tapering into very thin neck, 2–5 per metula, 10.5–23.5 × 2.0–3.5 μm; two types of conidia observed; microconidia subglobose to ellipsoidal, smooth-walled, hyaline, 3.5–4.5 × 2.0–4.0 μm; macroconidia subglobose, ellipsoidal to obovoid, smooth-walled, hyaline, 5.0–9.5 × 4.0–6.5 μm; chlamydospores subglobose, fusiform or irregular, smooth-walled, hyaline, 8.5–12 × 5.0–9.5 μm.

Notes: The new species appeared as a distinct lineage in ser. *Musarum* in the multi-locus and single-gene phylogenies (Figure 3 and Figures S7–S9). Morphologically, it differs from *T. musae* and *T. tiftonensis* of the same series in sticky colonies, the presence of chlamydospores, and two types of conidia [26,27]. The morphology of *T. ailsahockingiae* in the series was not given in the protologue [28] and thus could not be morphologically compared.

**Figure 8:**
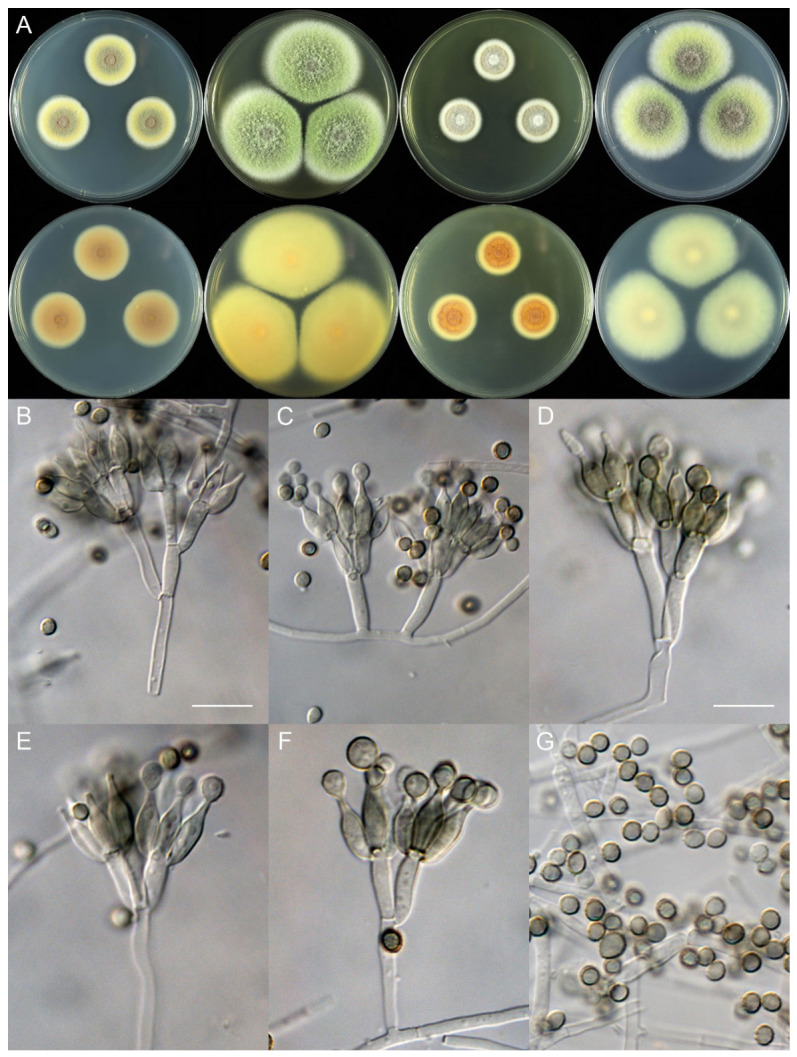
*Talaromyces heilongjiangensis* (HLJ58-02). (**A**) Colonies: top row left to right, obverse CYA, MEA, YES, and PDA; bottom row left to right, reverse CYA, MEA, YES, and PDA; (**B**–**F**) conidiophores; (**G**) conidia. Bars: (**B**) = 12.5 µm, also for (**C**); (**D**) = 10 µm, also for (**E**–**G**).

***Talaromyces heilongjiangensis*** X.C. Wang & W.Y. Zhuang, **sp. nov.** Figure 8

Fungal Names: FN571816

Etymology: The specific epithet refers to the type locality of the fungus.

In *Talaromyces* sect. *Talaromyces* ser. *Veerkampiorum*

Typification: CHINA. Heilongjiang Province, Jiamusi City, Fuyuan City, Nongjiang County, at the lakeside of Dalijia Lake, 48°16′52″ N 134°17′30″ E, in soil, 13 May 2023, Xin-Cun Wang and He Song, culture, He Song, HLJ58-02 (holotype HMAS 247930, preserved in a metabolically inactive state; ex-type strain CGMCC 3.29868).

DNA barcodes: ITS PP357621, *BenA* PP373072, *CaM* PP373077, *RPB2* PP373083.

Colony diam., 7 days, 25 °C (unless stated otherwise): CYA 24–27 mm; CYA 37 °C 27–32 mm; CYA 5 °C no growth; MEA 45–52 mm; YES 21–23 mm; PDA 31–43 mm.

Colony characteristics: On CYA 25 °C, 7 days: Colonies nearly circular, protuberant, concave at centers; margins moderately wide, entire; mycelia white and yellow; texture velutinous; sporulation moderately dense; conidia en masse yellowish green to greyish green; soluble pigments absent; exudates absent; reverse yellow to orange.

On CYA 37 °C, 7 days: Colonies nearly circular or irregular, plain or slightly protuberant, with radially sulcate or not; margins moderately narrow, entire; mycelia white; texture velutinous; sporulation absent or moderately dense; conidia en masse yellowish green; soluble pigments absent; exudates absent; reverse buff to light brown.

On MEA 25 °C, 7 days: Colonies nearly circular, plain; margins wide, entire or fimbriate; mycelia white; texture floccose; sporulation moderately dense to dense; conidia en masse yellowish green to greyish green; soluble pigments absent; exudates absent; reverse white to yellow.

On YES 25 °C, 7 days: Colonies nearly circular, protuberant, concentrically sulcate, concave at centers; margins moderately wide, fimbriate; mycelia white; texture velutinous to floccose; sporulation moderately dense to dense; conidia en masse brownish grey to greyish green; soluble pigments absent; exudates absent; reverse buff to orange.

On PDA 25 °C, 7 days: Colonies nearly circular or irregular, protuberant at centers; margins moderately wide to wide, entire or irregular; mycelia white; texture floccose; sporulation moderately dense to dense; yellowish green to grayish green conidia *en masse*; soluble pigments absent; exudates absent; reverse white or yellow to orange.

Micromorphology: Conidiophores biverticillate or terverticillate; stipes smooth-walled, 20–185 × 2.0–3.5 μm; rami 2, 10.5–17.5 × 2.5–3.5 μm; metulae 2–5, 8.5–13 × 2.5–4.5 μm; phialides ampulliform, tapering into very thin neck, 3–6 per metula, 8–12 × 3.5–4.5 μm; conidia subglobose to ellipsoidal, rough-walled, brown, 3.0–4.5 (–5.5) × 3.0–4.0 (–5.0) μm.

Additional strain examined: CHINA. Heilongjiang Province, Jiamusi City, Fuyuan City, Nongjiang County, at the lakeside of Dalijia Lake, 48°18′0″ N 134°17′23″ E, in soil, 13 May 2023, Xin-Cun Wang and He Song, culture, He Song, HLJ58-14.

Notes: This species is the member of ser. *Veerkampiorum* and phylogenetically related to *T. californicus*, *T. louisianensis* and *T. veerkampii* (Figure 6). It differs from *T. californicus* in 3 bp for *BenA*, 3 bp for *CaM* and 7 bp for *RPB2*; from *T. louisianensis* in 4 bp for *BenA*, 13 bp for *CaM*, and 4 bp for *RPB2*; and from *T. veerkampii* in 6 bp for *BenA*, 5 bp for *CaM* and 8 bp for *RPB2*. Morphologically, it differs from *T. californicus* in slower growth rate on CYA at 37 °C, terverticillate conidiophores and smaller conidia (3.0–4.5 × 3.0–4.0 vs. 4.0–6.0 × 4.0–7.0 μm) [29]; from *T. louisianensis* in slower growth rates on CYA at 25 °C and 37 °C and terverticillate conidiophores [29]; and from *T. veerkampii* in faster growth rates on CYA at 37 °C and on MEA at 25 °C, slower growth rate on YES, reverse buff to orange instead of dark green on YES, terverticillate conidiophores, broader phialides (3.5–4.5 vs. 3.0–3.5 μm wide), and rough-walled conidia [30]. Their morphological distinctions were summarized in Table 8.

**Figure 9:**
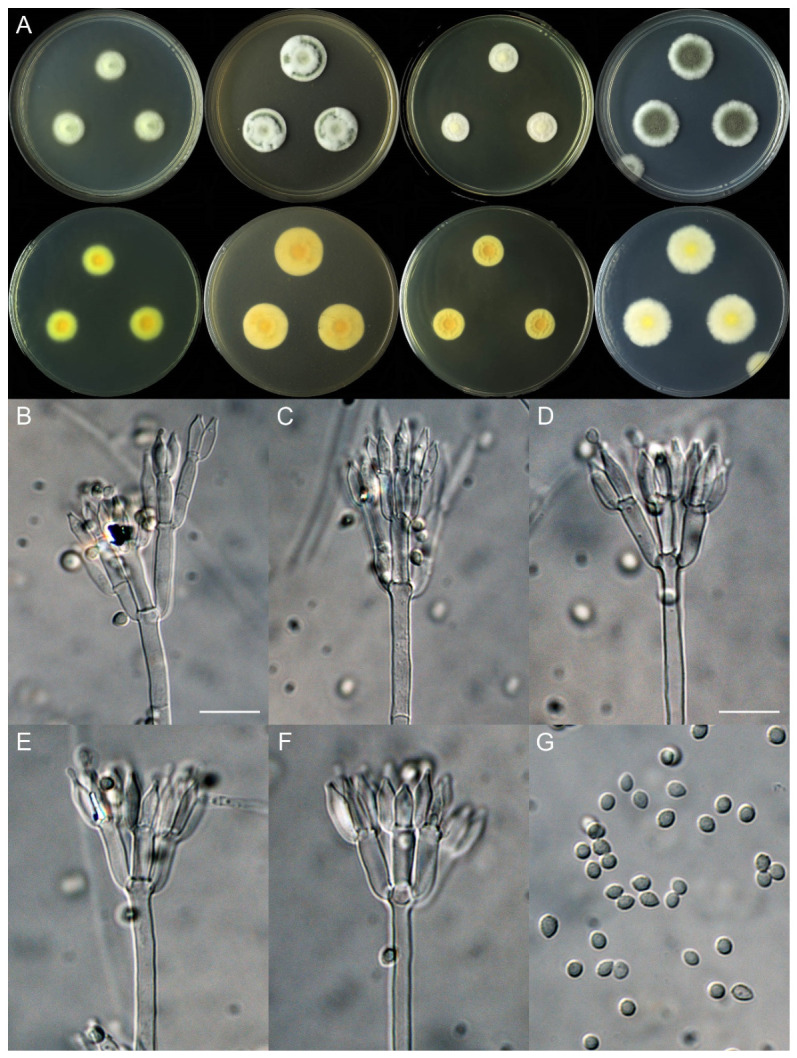
*Talaromyces tapisciae* (YN23-08). (**A**) Colonies: top row left to right, obverse CYA, MEA, YES, and PDA; bottom row left to right, reverse CYA, MEA, YES, and PDA; (**B**–**F**) conidiophores; (**G**) conidia. Bars: (**B**) = 12.5 µm, also for (**C**); (**D**) = 10 µm, also for (**E**–**G**).

***Talaromyces tapisciae*** X.C. Wang, L.Y. Peng & W.Y. Zhuang, **sp. nov.** Figure 9

Fungal Names: FN573765

Etymology: The specific epithet refers to the host plant of the fungus, *Tapiscia yunnanensis* W.C. Cheng & C.D. Chu.

In *Talaromyces* sect. *subinflati* ser. *subinflati*

Typification: CHINA. Yunnan Province, Xishuangbanna Dai Autonomous Prefecture, Mengla County, Mengla Town, Bubang Village, Wangtianshu (*Parashorea chinensis* H. Wang) Scenic Area, 21°37′24″ N 101°35′21″ E, in soil under rotten stump of *Tapiscia yunnanensis*, 31 May 2024, Xin-Cun Wang, culture, Lu-Yao Peng, YN23-08 (holotype HMAS 354376, preserved in a metabolically inactive state; ex-type strain CGMCC 3.29869).

DNA barcodes: ITS PZ326304, *BenA* PZ321404, *CaM* PZ321408, *RPB2* PZ321414.

Colony diam., 7 days, 25 °C (unless stated otherwise): CYA 17–18 mm; CYA 37 °C no growth; CYA 5 °C no growth; MEA 21–25 mm; YES 10–15 mm; PDA 22–26 mm.

Colony characteristics: On CYA 25 °C, 7 days: Colonies nearly circular, protuberant at centers; margins moderately wide, entire; mycelia white; texture velutinous; sporulation sparse; conidia en masse light grey; soluble pigments absent; exudates absent; reverse white to yellow.

On MEA 25 °C, 7 days: Colonies nearly circular, protuberant at centers; margins moderately wide to wide, entire; mycelia white; texture velutinous; sporulation moderately dense; conidia en masse yellowish green to greyish green; soluble pigments absent; exudates absent; reverse white to yellow.

On YES 25 °C, 7 days: Colonies nearly circular, slightly protuberant at centers, concentrically and radially sulcate; margins narrow, entire; mycelia white; texture velutinous; sporulation absent; soluble pigments absent; exudates absent; reverse white to yellow.

On PDA 25 °C, 7 days: Colonies nearly circular or irregular, plain; margins moderately wide to wide, entire or irregular; mycelia white; texture velutinous; sporulation dense; conidia en masse greenish grey; soluble pigments absent; exudates absent; reverse white, yellow at centers.

Micromorphology: Conidiophores biverticillate, in a minor portion terverticillate, rarely quaterverticillate; stipes smooth-walled, 250–725 × 3.0–4.5 μm; branches 2, 14–17.5 × 3.5–4.0 μm; rami 2–4, 8.5–19.5 × 3.5–4.5 μm; metulae 5–7, (8.5–) 11–15 (–17.5) × 3.5–4.5 μm; phialides ampulliform to acerose, tapering into very thin neck, 2–6 per metula, 8.5–11 (–15) × 2.5–3.5 μm; conidia subglobose to ellipsoidal, rough-walled, 3.0–4.0 (–6.5) × 2.5–3.0 (–4.0) μm.

Additional strain examined: CHINA. Yunnan Province, Xishuangbanna Dai Autonomous Prefecture, Mengla County, Mengla Town, Bubang Village, Wangtianshu (*Parashorea chinensis* H. Wang) Scenic Area, 21°37′24″ N 101°35′21″ E, in soil under rotten stump of *Tapiscia yunnanensis*, 31 May 2024, Xin-Cun Wang, culture, Lu-Yao Peng, YN23-06.

Notes: This species is sister to *T. jiangxiensis* with strong supports in the phylogenies inferred from combined and single-gene datasets (Figure 5 and Figures S13–S15). Molecularly, their differences include 5 bp for *BenA*, 18 bp for *CaM*, and 10 bp for *RPB2*. Morphologically, the new species differs from its sister in entire colonial margins on MEA 25 °C, terverticillate and quaterverticillate conidiophores, and rough-walled but not spiny conidia [31].

### 4.3. New Chinese Records

*Talaromyces angelicae* S.H. Yu, T.J. An & H.K. Sang, J. Microbiol. 51(5): 707, 2013.

In *Talaromyces* sect. *Talaromyces* ser. *Angelicarum*

Strain examined: CHINA. Xinjiang Uygur Autonomous Region, Changji Hui Autonomous Prefecture, Changji City, Liuhuanggou Town, 43°44′45″ N 87°13′12″ E, in soil, September 2015, Kai Chen, XJ6-2.

Notes: This species was first reported in South Korea and isolated from a medicinal plant *Angelica gigas* Nakai [32]. The Chinese strain is similar to the type strain of the fungus morphologically, but differs from the ex-type culture in 3 bp for the *RPB2* gene.

*Talaromyces gautengensis* Visagie & Yilmaz, Persoonia 53: 54, 2024.

In *Talaromyces* sect. *Talaromyces* ser. *Pinophili*

Strain examined: CHINA. Hebei Province, Handan City, Daming County, Daming Town, Youfentan Village, 36°17′37″ N 115°8′43″ E, in soil, 22 July 2023, Xin-Cun Wang, culture, Yi-Fan Wang, JJJ45-29.

Notes: This species was described from South Africa and had not been reported otherwhere [33]. The Chinese strain was identical to the ex-type culture in ITS and *BenA* sequences but having 5 bp differences for the *RPB2* gene. The Chinese material extends its distribution to Asia.

*Talaromyces rogersiae* Jurjević & S.W. Peterson, Mycologia 109(4): 550, 2017.

In *Talaromyces* sect. *Islandici* ser. *Wortmanniorum*

Strain examined: CHINA. Beijing City, Chaoyang District, Institute of Microbiology, Chinese Academy of Sciences, 40°0′15″ N 116°22′59″ E, as culture contaminant, 16 January 2025, Lu-Yao Peng, XCW_SN569.

Notes: This species was originally isolated from maize seed of North Carolina, USA [27]. The Chinese strain is identical to the ex-type culture in ITS and *RPB2* sequences (Figure S9). The Chinese material extends its distribution to Asia.

## 5. Discussion

Phylogenies of *Talaromyces* were reconstructed section by section based on separate or concatenated multi-locus datasets (*BenA*, *CaM* and *RPB2*). Fifty series were accordingly classified into nine sections., i.e., one in sections *Brunneospori* and *Tenues*, two in sections *Bacillispori* and *Helici*, three in sect. *Subinflati*, four in sect. *Purpurei*, five in sections *Islandici* and *Trachyspermi*, and 27 in the speciose section *Talaromyces*. Among the fifty series recognized, forty were newly introduced in this study. The updated series-level taxonomy of *Talaromyces* will facilitate species identification. Three new species were described based on phylogenetical and morphological information, i.e., *T. fujianensis* sp. nov. in sect. *Islandici*, *T. heilongjiangensis* sp. nov. in sect. *Talaromyces*, and *T. tapisciae* sp. nov. in sect. *subinflati*. Additionally, three new Chinese records were reported: *T. angelicae*, *T. gautengensis* and *T. rogersiae*. The findings of the new species and new Chinese records reveal the high diversity of the genus in China.

The most difficult part of this study is how to classify series of sect. *Talaromyces*. Among the 27 newly established series in the section, ten are monotypic. The species numbers of other series usually vary from two to 17. The key arguments for recognizing a series are based on the considerations whether it is a monophyly and how it is related to its allies. For example, series *Aprici* is sister to ser. *Angelicarum* in the combined locus phylogeny (Figure 6), but the grouping was not supported by the individual *BenA* or *RPB2* analyses (Figures S16 and S18). Similarly, the sister relationship between ser. *Dispares* and ser. *Intermedii* in the multi-locus phylogeny did not show in the single-gene trees (Figure 6 and Figures S16–S18). Thus, these monotypic series could not be simply grouped because of phylogenetical stability and taxonomical operability. The current divisions at series level will benefit a more accurate species identification by means of phylogenetic analysis at a smaller scale.

China is rich in species diversity of *Talaromyces*. This country is usually divided into seven geographic divisions: North China, Northeast China, Northwest China, Central China, East China, South China, and Southwest China. Many *Talaromyces* species were discovered in different parts, e.g., *T. gautengensis* and *T. rogersiae* of this study from North China, *T. heilongjiangensis* and *T. heiheensis* from Northeast China [34], *T. angelicae* from Northwest China, *T. mangshanicus* from Central China [34], *T. fujianensis* from East China, *T. xishaensis* from South China [35], and *T. chongqingensis*, *T. elephas*, *T. ginkgonis*, *T. shilinensis*, *T. sinensis*, *T. tapisciae*, *T. wushanicus* and *T. xishuangbannaensis* from Southwest China [14,36,37]. Notably, Southwest China harbors the highest biodiversity of the genus, which is in accordance with the discoveries of a recent investigation on fungal taxonomy of China [38]. China has four of the 36 biodiversity hotspots in the world: Himalaya, Indo-Burma, Mountains of Central Asia, and Mountains of Southwest China [39]; more efforts are needed to explore the underestimated areas of the country.

The taxonomic framework of *Talaromyces* at series level has been proposed, which will benefit better understanding of the group. Nevertheless, it cannot be perfect, and modifications will be undoubtedly needed in future. Along with introductions of additional new species, the concepts of series provided in this work might be improved. Further, introduction of additionally informative locus from genomes or application of phylogenomic approach to phylogeny of the genus may update our knowledge of the concepts of the series.

## Figures and Tables

**Table 1:** Species and sequences of *Talaromyces* sect. *Bacillispori* used in phylogenetic analyses.

Species	Strain	Country	Substrate	ITS	*BenA*	*CaM*	*RPB2*
*T. bacillisporus* (Swift) C.R. Benj. 1955	CBS 296.48 T	North America	leaves of *Begonia*	KM066182	AY753368	KJ885262	JF417425
*T. clematidis* Spetik & Houbraken 2023	CBS 149228 T	Czech Republic	root of *Clematis*	ON863768	ON873763	ON938196	ON938200
*T. columbiensis* N. Yilmaz et al. 2016	CBS 113151 T	Colombia	leaf litter	KX011503	KX011488	KX011499	MN969187
*T. cupressi* V. Meshram et al. 2022	CBS 147104 T	Israel	*Phloeosinus bicolor* colonizing *Cupressus sempervirens*	MT955352	MT991527	MT991517	MT991522
*T. emodensis* Udagawa 1993	CBS 100536 T	Nepal	paddy soil	JN899337	KJ865724	KJ885269	JF417445
*T. maltbyae* Y.P. Tan et al. 2024	MST FP2571 T	Australia	soil	PP665725	PP682577	PP682548	PP682564
*T. mimosinus* A.D. Hocking 1980	CBS 659.80 T	Australia	soil	JN899338	KJ865726	KJ885272	MN969149
*T. proteolyticus* (Kamyschko) Samson et al. 2011	CBS 303.67 T	Russia	soil	JN899387	KJ865729	KJ885276	KM023301
*T. unicus* Tzean et al. 1992	CBS 100535 T	China: Taiwan	soil	JN899336	KJ865735	KJ885283	MN969150
*T. subinflatus* Yaguchi & Udagawa 1993	CBS 652.95 T	Japan	soil	JN899397	MK450890	KJ885280	KM023308

**Table 2:** Species and sequences of sections *Brunneospori*, *Helici* and *Tenues* in *Talaromyces* used in phylogenetic analyses.

Species	Strain	Country	Substrate	ITS	*BenA*	*CaM*	*RPB2*
*T. aerugineus* (Samson) N. Yilmaz et al. 2014	CBS 350.66 T	UK	swamp	AY753346	KJ865736	KJ885285	JN121502
*T. bohemicus* (Fassat. & Pěčková) N. Yilmaz et al. 2014	CBS 545.86 T	Czech Republic	peat	JN899400	KJ865719	KJ885286	JN121532
*T. boninensis* (Yaguchi & Udagawa) Samson et al. 2011	CBS 650.95 T	Japan	soil	JN899356	KJ865721	KJ885263	KM023276
*T. borbonicus* Houbraken 2018	CBS 141340 T	Italy	decayed *Arundo donax*	MG827091	MG855687	MG855688	MG855689
*T. cinnabarinus* (S.C. Jong & E.E. Davis) N. Yilmaz et al. 2014	CBS 267.72 T	Japan	pepper field soil	JN899376	AY753377	KJ885256	JN121477
*T. diversiformis* A.J. Chen et al. 2016	CGMCC 3.18204 T	China: Beijing	indoor air	KX961215	KX961216	KX961259	KX961274
*T. georgiensis* Guevara-Suarez et al. 2017	CBS 142380 T	USA	animal joint fluid	LT558967	LT559084	n.a.	LT795606
*T. helicus* (Raper & Fennel) C.R. Benj. 1955	CBS 335.48 T	Sweden	soil	JN899359	KJ865725	KJ885289	KM023273
*T. koreanus* Hyang B. Lee 2021	CNUFC YJW2-13 T	South Korea	freshwater	MZ315100	MZ318450	MZ332529	MZ332533
*T. pigmentosus* R.N. Barbosa et al. 2018	CBS 142805 T	Brazil	nest of *Melipona scutellaris*	MF278330	LT855562	LT855565	LT855568
*T. reverso-olivaceus* A.J. Chen et al. 2016	CGMCC 3.18195 T	China: Beijing	indoor air	KU866646	KU866834	KU866730	KU866990
*T. tabacinus* Jurjević et al. 2018	NRRL 66727 T	USA	leaves of *Nicotiana tabacum*	MG182613	MG182627	MG182606	MG182620
*T. teleomorphus* Hyang B. Lee et al. 2021	CNUFC YJW2-5 T	South Korea	freshwater	MZ315102	MZ318452	MZ332531	MZ332535
*T. varians* (G. Sm.) Samson et al. 2011	CBS 386.48 T	UK	cotton yarn	JN899368	KJ865731	KJ885284	KM023274
*T. brunneosporus* Rodr.-Andr. et al. 2019	CBS 144320 T	Spain	honey	LT962487	LT962483	LT962488	LT962485
*T. tenuis* B.D. Sun et al. 2020	CBS 141840 T	China: Guizhou	soil	MN864275	MN863344	MN863321	MN863333

“n.a.” is the abbreviation for “not available”.

**Table 3:** Species and sequences of *Talaromyces* sect. *Islandici* used in phylogenetic analyses.

Species	Strain	Country	Substrate	ITS	*BenA*	*CaM*	*RPB2*
*T. acaricola* Visagie et al. 2016	CBS 137386 T	South Africa	Acari associated with infructescence of *Protea repens*	JX091476	JX091610	JX140729	KF984956
*T. ailsahockingiae* Y.P. Tan et al. 2024	MST FP2620 T	Australia	*Homo sapiens*	PP416843	PP438386	PP438370	PP438379
*T. allahabadensis* (B.S. Mehrotra & D. Kumar) Samson et al. 2011	CBS 453.93 T	India	cultivated soil	KF984873	KF984614	KF984768	KF985006
*T. atricola* (Thom) S.W. Peterson & Jurjević 2013	CBS 255.31 T	unknown	unknown	KF984859	KF984566	KF984719	KF984948
*T. brunneus* (Udagawa) Samson et al. 2011	CBS 227.60 T	Thailand	milled *Oryza sativa*	JN899365	KJ865722	KJ885264	KM023272
*T. cerinus* A.J. Chen et al. 2016	CGMCC 3.18212 T	China: Beijing	indoor air	KU866658	KU866845	KU866742	KU867002
*T. chlamydosporus* A.J. Chen et al. 2016	CGMCC 3.18199 T	China: Beijing	indoor air	KU866648	KU866836	KU866732	KU866992
*T. columbinus* S.W. Peterson & Jurjević 2013	NRRL 58811 T	USA	air	KJ865739	KF196843	KJ885288	KM023270
*T. crassus* Visagie et al. 2016	CBS 137381 T	South Africa	infructescence of *Protea repens*	JX091472	JX091608	JX140727	KF984914
*T. delawarensis* Jurjević & S.W. Peterson 2017	NRRL 58874 T	USA	indoor air sample	KX657324	KX657055	KX657158	KX657490
*T. endophyticus* L. Su & Y.C. Niu 2018	ACCC 39141 T	China: Shandong	stems of *Cucumis sativus*	KX639168	KX639174	KX639165	n.a.
***T. fujianensis* X.C. Wang, L.Y. Peng & W.Y. Zhuang, sp. nov.**	**FJ12-14 T**	China: Fujian	soil	**PZ326302**	**PZ321402**	**PZ321406**	**PZ321412**
*T. guiyangensis* Zhi.Y. Zhang et al. 2023	CGMCC 3.20782 T	China: Guizhou	soil	OL897027	ON569046	ON568886	ON568965
*T. herodensis* Jurjević & S.W. Peterson 2017	NRRL 62467 T	USA	seed of *Arachis hypogaea*	KX657338	KX657061	KX657182	KX657524
*T. infraolivaceus* Visagie et al. 2016	CBS 137385 T	South Africa	Acari associated with infructescence of *Protea repens*	JX091481	JX091615	JX140734	KF984949
*T. islandicus* (Sopp) Samson et al. 2011	CBS 338.48 T	South Africa	unknown	KF984885	KF984655	KF984780	KF985018
*T. juglandicola* Jurjević & S.W. Peterson 2017	NRRL 32382 T	USA	decaying fruit husk of *Juglans nigra*	KX657330	KX657122	KX657184	KX657573
*T. kilbournensis* Jurjević & S.W. Peterson 2017	NRRL 62700 T	USA	Nitidulidae	KX657344	KX657068	KX657183	KX657545
*T. loliensis* (Pitt) Samson et al. 2011	CBS 643.80 T	New Zealand	*Lolium*	KF984888	KF984658	KF984783	KF985021
*T. musae* Houbraken et al. 2017	CBS 142504 T	Germany	tip of banana	MF072316	MF093729	MF093728	MF093727
*T. neorugulosus* A.J. Chen et al. 2016	CGMCC 3.18215 T	China: Beijing	indoor air	KU866659	KU866846	KU866743	KU867003
*T. novojersensis* Jurjević & S.W. Peterson 2017	NRRL 35858 T	USA	indoor air	KX657319	KX657050	KX657151	KX657503
*T. piceus* (Raper & Fennell) Samson et al. 2011	CBS 361.48 T	unknown	unknown	KF984792	KF984668	KF984680	KF984899
*T. podocarpi* Visagie & Yilmaz 2024	CBS 152015 T	South Africa	soil	PP375126	PP356399	PP356461	PP356494
*T. pseudorugulosus* Q.M. Wang et al. 2025	CGMCC 3.16296 T	China: Xizang	soil	ON427037	ON667682	ON703619	ON703699
*T. radicus* (A.D. Hocking & Whitelaw) Samson et al. 2011	CBS 100489 T	Australia	root of seedling *Triticum aestivum*	JN899324	KF984599	KF984773	KF985013
*T. ricevillensis* Jurjević & S.W. Peterson 2017	NRRL 62296 T	USA	swine feed	KX657343	KX657056	KX657249	KX657582
*T. rogersiae* Jurjević & S.W. Peterson 2017	NRRL 62223 T	USA	seed of *Zea mays*	KX657332	KX657125	KF196891	KX657581
	**XCW_SN569**	China: Beijing	culture contaminant	**PZ326301**	n.a.	n.a.	**PZ321411**
*T. rotundus* (Raper & Fennell) C.R. Benj. 1955	CBS 369.48 T	Panama	wood	JN899353	KJ865730	KJ885278	KM023275
*T. rugulosus* (Thom) Samson et al. 2011	CBS 371.48 T	USA	rotting potato tubers	KF984834	KF984575	KF984702	KF984925
*T. scorteus* (Nakaz. et al.) S.W. Peterson & Jurjević 2013	CBS 340.34 T	Japan	military equipment	KF984892	KF984565	KF984684	KF984916
*T. siglerae* S.W. Peterson & Jurjević 2017	NRRL 28620 T	Canada	tinea capitis infection of *Homo sapiens*	KX657351	KX657135	KX657236	KX657497
*T. subaurantiacus* Visagie et al. 2016	CBS 137383 T	South Africa	fynbos soil	JX091475	JX091609	JX140728	KF984960
*T. subtropicalis* Jurjević & S.W. Peterson 2017	NRRL 58084 T	USA	air sample	KX657337	KX657060	KX657250	KX657531
*T. tardifaciens* Udagawa 1993	CBS 250.94 T	Nepal	paddy soil	JN899361	KF984560	KF984682	KF984908
*T. tiftonensis* Jurjević & S.W. Peterson 2017	NRRL 62264 T	USA	seed of *Zea mays*	KX657353	KX657129	KX657163	KX657602
*T. tratensis* Manoch et al. 2013	CBS 133146 T	Thailand	forest soil	KF984891	KF984559	KF984690	KF984911
*T. variabilis* (Sopp) Samson et al. 2011	CBS 385.48 T	South Africa	coconut matting	JN899343	JX494295	n.a.	n.a.
*T. wortmanii* (Klöcker) C.R. Benj. 1955	CBS 391.48 T	Denmark	soil	KF984829	KF984648	KF984756	KF984977
*T. yelensis* Visagie et al. 2014	CBS 138209 T	Micronesia	house dust	KJ775717	KJ775210	KP119161	KP119163
*T. bacillisporus* (Swift) C.R. Benj. 1955	CBS 296.48 T	North America	leaves of *Begonia*	KM066182	AY753368	KJ885262	JF417425

GenBank accession numbers in bold indicate the newly generated sequences. “n.a.” is the abbreviation for “not available”.

**Table 4:** Species and sequences of *Talaromyces* sect. *Purpurei* used in phylogenetic analyses.

Species	Strain	Country	Substrate	ITS	*BenA*	*CaM*	*RPB2*
*T. cattleyae* T.O. Condé et al. 2025	COAD 3659 T	Brazil	healthy roots of *Cattleya locatellii*	PP90542	PP941896	PP941887	PP941892
*T. cecidicola* (Seifert et al.) Samson et al. 2011	CBS 101419 T	USA	galls of Cynipidae on twigs of *Quercus pacifica*	AY787844	FJ753295	KJ885287	KM023309
*T. chlorolomus* Visagie & K. Jacobs 2012	DAOM 241016 T	South Africa	fynbos soil	FJ160273	GU385736	KJ885265	KM023304
*T. coalescens* (Quintan.) Samson et al. 2011	CBS 103.83 T	Spain	soil	JN899366	JX091390	KJ885267	KM023277
*T. dendriticus* (Pitt) Samson et al. 2011	CBS 660.80 T	Australia	leaf litter of *Eucalyptus pauciflora*	JN899339	JX091391	KF741965	KM023286
*T. freemaniae* Y.P. Tan et al. 2024	MST FP2577 T	Australia	bark of *Allocasuarina* sp.	PP665726	PP682578	PP682549	PP682565
*T. gwangjuensis* Hyang B. Lee & T.T.T. Nguyen 2021	CNUFC WT19-1 T	South Korea	freshwater	MK766233	MZ318448	n.a.	MK912174
*T. ignescens* Van Vuuren et al. 2025	CBS 153397 T	South Africa	soil	MH281565	PV550672	PV550673	PV550674
*T. iowaensis* Jurjević et al. 2018	ITEM 17527 T	USA	office air	MH281565	MH282578	MH282579	MH282577
*T. macrodendroideus* Visagie et al. 2024	PPRI 16060 T	South Africa	unknown	MK450749	MK451204	MK451692	MK450886
*T. mzansiensis* Visagie et al. 2024	PPRI 3887 T	South Africa	unknown	MK450748	MK451184	MK451691	MK450885
*T. pittii* (Quintan.) Samson et al. 2011	CBS 139.84 T	Spain	clayey soil	JN899325	KJ865728	KJ885275	KM023297
*T. pseudostromaticus* (Hodges et al.) Samson et al. 2011	CBS 470.70 T	USA	feathers of *Hylocichla fuscescens*	JN899371	HQ156950	KJ885277	KM023298
*T. ptychoconidius* Visagie & K. Jacobs 2012	DAOM 241017 T	South Africa	fynbos soil	FJ160266	GU385733	JX140701	KM023278
*T. pulveris* Crous 2020	CBS 146831 T	France	bore dust of *Xestobium rufovillosum*	MW175345	MW173136	MW173099	MW173115
*T. purpureus* (E. Müll. & Pacha-Aue) Stolk & Samson 1972	CBS 475.71 T	France	soil	JN899328	GU385739	KJ885292	JN121522
*T. rademirici* (Quintan.) Samson et al. 2011	CBS 140.84 T	Spain	air	JN899386	KJ865734	n.a.	KM023302
*T. ramulosus* (Visagie & K. Jacobs) Samson et al. 2011	DAOM 241660 T	South Africa	fynbos soil	EU795706	FJ753290	JX140711	KM023281
*T. rickardiae* Y.P. Tan et al. 2024	MST FP2588 T	Australia	bark of *Grevillea striata*	PP665727	PP682579	PP682550	PP682566
*T. saxoxalicus* J. Trovão et al. 2021	MUM 20.30 T	Portugal	biofilm covering deteriorated limestone wall	MT039882	MT052003	n.a.	MT052004
*T. trachyspermus* (Shear) Stolk & Samson 1972	CBS 373.48	USA	unknown	JN899354	KF114803	KJ885281	JF417432

“n.a.” is the abbreviation for “not available”.

**Table 5:** Species and sequences of *Talaromyces* sect. *Subinflati* used in phylogenetic analyses.

Species	Strain	Country	Substrate	ITS	*BenA*	*CaM*	*RPB2*
*T. guizhouensis* B.D. Sun et al. 2020	CBS 141837 T	China: Guizhou	soil	MN864277	MN863346	MN863323	MN863335
*T. jiangxiensis* Zhi.Y. Zhang et al. 2023	CGMCC 3.20783 T	China: Jiangxi	soil	OL897029	ON569044	ON568888	ON568963
*T. paecilomycetoides* Zhi.Y. Zhang et al. 2023	CGMCC 3.20785 T	China: Yunnan	soil	OL897033	ON569040	ON568890	ON568959
*T. palmae* (Samson et al.) Samson et al. 2011	CBS 442.88 T	Netherlands	seeds of *Chrysalidocarpus lutescens*	JN899396	HQ156947	KJ885291	KM023300
*T. parapalmae* Zhi Y. Zhang & Y.F. Han 2024	CGMCC 3.25510 T	China: Guizhou	soil	OR680520	OR843225	OR828456	OR842937
*T. resedanus* (McLennan & Ducker) A.J. Chen et al. 2020	CBS 181.71 T	Australia	acid, sandy soil	MN431413	MN969436	MN969355	MN969214
*T. sinensis* X.C. Wang et al. 2025	CGMCC 3.28744 T	China: Yunnan	rotten husk of an unknown fruit	PV085755	PV102705	PV102718	PV102726
*T. subinflatus* Yaguchi & Udagawa 1993	CBS 652.95 T	Japan	soil	JN899397	MK450890	KJ885280	KM023308
***T. tapisciae* X.C. Wang, L.Y. Peng & W.Y. Zhuang, sp. nov.**	**YN23-08 T**	China: Yunnan	fallen rotten tree of *Tapiscia yunnanensis*	**PZ326304**	**PZ321404**	**PZ321408**	**PZ321414**
	**YN23-06**	China: Yunnan	fallen rotten tree of *Tapiscia yunnanensis*	**PZ326303**	**PZ321403**	**PZ321407**	**PZ321413**
*T. tzapotlensis* Jurjević & S.W. Peterson 2017	NRRL 35203 T	Mexico	*Hypothenemus hampei*	KX946902	KX946884	KX946893	KX946922
*T. bacillisporus* (Swift) C.R. Benj. 1955	CBS 296.48 T	North America	leaves of *Begonia*	KM066182	AY753368	KJ885262	JF417425

GenBank accession numbers in bold indicate the newly generated sequences.

**Table 6:** Species and sequences of *Talaromyces* sect. *Talaromyces* used in phylogenetic analyses.

Species	Strain	Country	Substrate	ITS	*BenA*	*CaM*	*RPB2*
*T. aculeatus* (Raper & Fennell) Samson et al. 2011	NRRL 2129 T	USA	weathering fabric	KF741995	KF741929	KF741975	MH793099
*T. adpressus* A.J. Chen et al. 2016	CGMCC 3.18211 T	China: Beijing	indoor air	KU866657	KU866844	KU866741	KU867001
*T. alveolaris* Guevara-Suarez et al. 2017	CBS 142379 T	USA	human bronchoalveolar lavage	LT558969	LT559086	LT795596	LT795597
*T. amazonensis* N. Yilmaz et al. 2016	CBS 140373 T	Colombia	leaf litter	KX011509	KX011490	KX011502	MN969186
*T. amestolkiae* N. Yilmaz et al. 2012	CBS 132696 T	South Africa	house dust	JX315660	JX315623	KF741937	JX315698
*T. angelicae* S.H. Yu et al. 2013	KACC 46611 T	South Korea	dried root of *Angelica gigas*	KF183638	KF183640	KJ885259	KX961275
	**XJ6-2**	China: Xinjiang	soil	**PZ326299**	**PZ321400**	**PZ321405**	**PZ321409**
*T. annesophieae* Houbraken 2017	CBS 142939 T	Netherlands	soil	MF574592	MF590098	MF590104	MN969199
*T. apiculatus* Samson et al. 2011	CBS 312.59 T	Japan	soil	JN899375	KF741916	KF741950	KM023287
*T. apricus* Y.P. Tan et al. 2024	MST FP2583 T	USA	soil	PP665730	PP682582	PP682553	PP682569
*T. argentinensis* Jurjević & S.W. Peterson 2019	NRRL 28750 T	Ghana	soil	MH793045	MH792917	MH792981	MH793108
*T. aspriconidius* B.D. Sun et al. 2020	CBS 141835 T	China: Yunnan	soil	MN864274	MN863343	MN863320	MN863332
*T. astoniae* Tan et al. 2024	MST FP2622 T	Australia	soil	PP416844	PP438387	PP438371	PP438380
*T. atkinsoniae* Y.P. Tan et al. 2022	BRIP 72528s T	Australia	gills of *Marasmius crinisequi*	OP059084	OP087524	n.a.	OP087523
*T. aurantiacus* (J.H. Mill. et al.) Samson et al. 2011	CBS 314.59 T	USA	nursery soil	JN899380	KF741917	KF741951	KX961285
*T. aureolinus* L. Wang 2021	CGMCC 3.15865 T	China: Yunnan	soil	MK837953	MK837937	MK837945	MK837961
*T. australis* Visagie et al. 2015	CBS 137102 T	Australia	soil under pasture	KF741991	KF741922	KF741971	KX961284
*T. bannicus* L. Wang 2021	CGMCC 3.15862 T	China: Yunnan	soil	MK837955	MK837939	MK837947	MK837963
*T. beariae* Tan et al. 2024	MST FP2623 T	Australia	soil	PP416845	PP438388	PP438372	PP438381
*T. beijingensis* A.J. Chen et al. 2016	CGMCC 3.18200 T	China: Beijing	indoor air	KU866649	KU866837	KU866733	KU866993
*T. benedictus* D.S. Paiva 2025	MUM 23.44 T	Portugal	limestone	PP151473	PP453634	PP453612	PP453642
*T. brevis* B.D. Sun et al. 2020	CBS 141833 T	China: Beijing	soil	MN864269	MN863338	MN863315	MN863328
*T. calidicanius* (J.L. Chen) Samson et al. 2011	CBS 112002 T	China: Taiwan	soil	JN899319	HQ156944	KF741934	KM023311
*T. californicus* Jurjević & S.W. Peterson 2019	NRRL 58168 T	USA	air	MH793056	MH792928	MH792992	MH793119
*T. cavernicola* V.C.S. Alves et al. 2022	URM 8448 T	Brazil	air in cave	ON862935	OP672383	OP290543	OP290515
*T. cnidii* S.H. Yu et al. 2013	KACC 46617 T	South Korea	dried roots of *Cnidium officinale*	KF183639	KF183641	KJ885266	KM023299
*T. coprophilus* M. Guevara-Suarez et al. 2020	CBS 142756 T	Spain	herbivore dung	LT899794	LT898319	LT899776	LT899812
*T. cucurbitiradicus* L. Su & Y.C. Niu 2018	ACCC 39155 T	China: Beijing	endophyte from root of *Cucurbita moschata*	KY053254	KY053228	KY053246	n.a.
*T. derxii* Takada & Udagawa 1988	CBS 412.89 T	Japan	cultivated soil	JN899327	JX494306	KF741959	KM023282
*T. dimorphus* X.Z. Jiang & L. Wang 2018	CGMCC 3.15692 T	China: Hainan	forest soil	KY007095	KY007111	KY007103	KY112593
*T. disparis* Y. Ruan & L. Wang 2024	CGMCC 3.26221 T	China: Hainan	soil	PP544888	PP566271	PP566276	PP555175
*T. doitungensis* Thakshila et al. 2026 (*Talaromyces* sp. MFLUCC 24-0321)	MFLUCC 24-0321 T	Thailand	soil	PQ325260	PQ330891	n.a.	PQ330892
*T. domesticus* Jurjević & S.W. Peterson 2019	NRRL 58121 T	USA	floor swab	MH793055	MH792927	MH792991	MH793118
*T. duclauxii* (Delacr.) Samson et al. 2011	CBS 322.48 T	France	canvas	JN899342	JX091384	KF741955	JN121491
*T. echinulatus* Hyang B. Lee & T.T.T. Nguyen 2023	CNUFC HB1206 T	South Korea	soil	OR462362	OR507571	OR608367	OR591610
*T. euchlorocarpius* Yaguchi et al. 1999	CBM PF1203 T	Japan	soil	AB176617	KJ865733	KJ885271	KM023303
*T. exleyae* Y.P. Tan et al. 2024	MST FP2568 T	Australia	soil	PP665731	PP682583	PP682555	PP682570
*T. flavovirens* (Durieu & Mont.) Visagie et al. 2012	CBS 102801 T	Spain	dead leaves of *Quercus ilex*	JN899392	JX091376	KF741933	KX961283
*T. flavus* (Klöcker) Stolk & Samson 1972	CBS 310.38 T	New Zealand	unknown	JN899360	JX494302	KF741949	JF417426
*T. francoae* N. Yilmaz et al. 2016	CBS 113134 T	Colombia	leaf litter	KX011510	KX011489	KX011501	MN969188
*T. funiculosus* (Thom) Samson et al. 2011	CBS 272.86 T	India	*Lagenaria vulgaris*	JN899377	MN969408	KF741945	KM023293
*T. funiformis* Y. Ruan & L. Wang 2024	CGMCC 3.26220 T	China: Hainan	soil	PP544886	PP566269	PP566274	PP555173
*T. fuscoviridis* Visagie et al. 2015	CBS 193.69 T	Netherlands	soil	KF741979	KF741912	KF741942	MN969156
*T. fusiformis* A.J. Chen et al. 2016	CGMCC 3.18210 T	China: Beijing	indoor air	KU866656	KU866843	KU866740	KU867000
*T. galapagensis* Samson & Mahoney 1977	CBS 751.74 T	Ecuador	soil under *Maytenus obovata*	JN899358	JX091388	KF741966	KX961280
*T. gautengensis* Visagie & Yilmaz 2024	PPRI 25904 T	South Africa	soil	MK450750	MK451099	MK451693	MK450887
	**JJJ45-29**	China: Hebei	soil	**PZ326300**	**PZ321401**	n.a.	**PZ321410**
*T. ginkgonis* X.C. Wang & W.Y. Zhuang 2022	CGMCC 3.20698 T	China: Sichuan	diseased fruit of *Ginkgo biloba*	OL638158	OL689844	OL689846	OL689848
*T. hainanensis* K. Hong & L. Liu 2024	WHUF0341 T	China: Hainan	mangrove root soil	ON564542	ON908368	ON908369	ON569094
*T. haitouensis* L. Wang 2022	CGMCC 3.16101 T	China: Jiangsu	riverside soil	MZ045695	MZ054634	MZ054637	MZ054631
***T. heilongjiangensis* X.C. Wang & W.Y. Zhuang, sp. nov.**	**HLJ58-02 T**	China: Heilongjiang	soil at the lakeside	**PP357621**	**PP373072**	**PP373077**	**PP373083**
	**HLJ58-14**	China: Heilongjiang	soil at the lakeside	**PP357622**	**PP373073**	**PP373078**	**PP373084**
*T. indigoticus* Takada & Udagawa 1993	CBS 100534 T	Japan	soil	JN899331	JX494308	KF741931	KX961278
*T. intermedius* (Apinis) Stolk & Samson 1972	CBS 152.65 T	UK	swamp soil	JN899332	JX091387	KJ885290	KX961282
*T. jianfengicus* Y. Ruan & L. Wang 2024	CGMCC 3.26253 T	China: Hainan	soil	PP544889	PP566272	PP566277	PP555176
*T. johnpittii* E. Lacey et al. 2024	BRIP 75204a T	Australia	soil	OP712677	OP712647	OP712645	OP712646
*T. kabodanensis* Houbraken et al. 2016	CBS 139564 T	Iran	hypersaline soil	KP851981	KP851986	KP851995	MN969190
*T. kendrickii* Visagie et al. 2015	CBS 136666 T	Canada	conifer lumber	KF741987	KF741921	KF741967	MN969158
*T. lentulus* X.Z. Jiang & L. Wang 2018	CGMCC 3.15689 T	China: Shandong	soil	KY007088	KY007104	KY007096	KY112586
*T. liani* (Kamyschko) N. Yilmaz et al. 2014	CBS 225.66 T	China	soil	JN899395	JX091380	KJ885257	KX961277
*T. linderae* Y.P. Tan et al. 2024	MST FP2582 T	Australia	soil	PP665732	PP682585	PP682557	PP682572
*T. louisianensis* Jurjević & S.W. Peterson 2019	NRRL 35823 T	USA	air	MH793052	MH792924	MH792988	MH793115
*T. macrosporus* (Stolk & Samson) Frisvad et al. 1990	CBS 317.63 T	South Africa	apple juice	JN899333	JX091382	KF741952	KM023292
*T. mae* X.Z. Jiang & L. Wang 2018	CGMCC 3.15690 T	China: Shanghai	forest soil	KY007090	KY007106	KY007098	KY112588
*T. malicola* Jurjević & S.W. Peterson 2019	NRRL 3724 T	Italy	rhizosphere of an apple tree	MH909513	MH909406	MH909459	MH909567
*T. mangshanicus* X.C. Wang & W.Y. Zhuang 2017	CGMCC 3.18013 T	China: Hunan	soil	KX447531	KX447530	KX447528	KX447527
*T. marneffei* (Segretain et al.) Samson et al. 2011	CBS 388.87 T	Vietnam	*Rhizomys sinensis*	JN899344	JX091389	KF741958	KM023283
*T. minnsiorum* Tan et al. 2023	MST FP22313 T	Australia	soil	OR731313	OR737778	OR737767	OR737772
*T. muroii* Yaguchi et al. 1994	CBS 756.96 T	China: Taiwan	soil	MN431394	KJ865727	KJ885274	KX961276
*T. mycothecae* R.N. Barbosa et al. 2018	CBS 142494 T	Brazil	nest of *Melipona scutellaris*	MF278326	LT855561	LT855564	LT855567
*T. nanjingensis* X.R. Sun et al. 2022	CCTCC M2012167 T	China: Jiangsu	rhizosphere soil of *Pinus massoniana*	MW130720	MW147759	MW147760	MW147762
*T. neofusisporus* L. Wang 2016	CGMCC 3.15415 T	China: Tibet	leaf sample	KP765385	KP765381	KP765383	MN969165
*T. oumae-annae* Visagie et al. 2014	CBS 138208 T	South Africa	house dust	KJ775720	KJ775213	KJ775425	KX961281
*T. panamensis* (Samson et al.) Samson et al. 2011	CBS 128.89 T	Panama	soil	JN899362	HQ156948	KF741936	KM023284
*T. penicillioides* L. Wang 2021	CGMCC 3.15822 T	China: Guizhou	soil	MK837956	MK837940	MK837948	MK837964
*T. perryae* Y.P. Tan et al. 2024	MST FP2580 T	Australia	plant-based substrates	PP665729	PP682581	PP682552	PP682568
*T. pinophilus* (Hedgc.) Samson et al. 2011	CBS 631.66 T	France	polyvinyl chloride plastic	JN899382	JX091381	KF741964	KM023291
*T. popeae* Y.P. Tan et al. 2024	MST FP2591 T	Australia	termite nest	PP665734	PP682587	PP682559	PP682574
*T. potiguarorum* J.M.S. Lima et al. 2024	URM 8664 T	Brazil	insectivorous bat guano	PP034175	PP150745	PP150753	PP187794
*T. pratensis* Jurjević & S.W. Peterson 2019	NRRL 62170 T	USA	effluent of water treatment plant	MH793075	MH792948	MH793012	MH793139
*T. primulinus* (Pitt) Samson et al. 2011	CBS 321.48 T	USA	unknown	JN899317	JX494305	KF741954	KM023294
*T. pseudofuniculosus* M. Guevara-Suarez et al. 2020	CBS 143041 T	Spain	herbivore dung	LT899796	LT898323	LT899778	LT899814
*T. purgamentorum* N. Yilmaz et al. 2016	CBS 113145 T	Colombia	leaf litter	KX011504	KX011487	KX011500	MN969189
*T. purpureogenus* (Stoll) Samson et al. 2011	CBS 286.36 T	Japan	culture contaminant	JN899372	JX315639	KF741947	JX315709
*T. qii* L. Wang 2016	CGMCC 3.15414 T	China: Tibet	leaf sample	KP765384	KP765380	KP765382	MN969164
*T. rapidus* Guevara-Suarez et al. 2017	CBS 142382 T	USA	human bronchoalveolar lavage	LT558970	LT559087	LT795600	LT795601
*T. rosorhizae* H. Zhang & Y.L. Jiang 2021	GUCC 190040.1 T	China: Guizhou	endophyte of *Rosa roxburghii*	MZ221603	MZ333143	MZ333137	MZ333141
*T. ruber* (Stoll) N. Yilmaz et al. 2012	CBS 132704 T	UK	aircraft fuel tank	JX315662	JX315629	KF741938	JX315700
*T. rubicundus* (J.H. Mill. et al.) Samson et al. 2011	CBS 342.59 T	USA	nursery soil	JN899384	JX494309	KF741956	KM023296
*T. rufus* B.D. Sun et al. 2020	CGMCC 3.13203 T	China: Yunnan	soil	MN864272	MN863341	MN863318	MN863331
*T. santanderensis* B.E. Guerra-Sierra & L.A. Arteaga-Figueroa 2022	HF05 T	Colombia	rhizosphere soil of *Theobroma cacao*	OP082331	OP067657	OP067656	OP067655
*T. sayulitensis* Visagie et al. 2014	CBS 138204 T	Mexico	house dust	KJ775713	KJ775206	KJ775422	MN969146
*T. shepherdshillensis* Y.P. Tan et al. 2024	MST FP3281 T	Australia	soil	PP416846	PP438389	PP438373	PP438382
*T. shilinensis* X.C. Wang & W.Y. Zhuang 2022	CGMCC 3.20699 T	China: Yunnan	ascomata of *Pseudocosmospora* sp.	OL638159	OL689845	OL689847	OL689849
*T. siamensis* (Manoch & C. Ramírez) Samson et al. 2011	CBS 475.88 T	Thailand	forest soil	JN899385	JX091379	KF741960	KM023279
*T. soli* Jurjević & S.W. Peterson 2019	NRRL 62165 T	USA	soil	MH793074	MH792947	MH793011	MH793138
*T. sparsus* L. Wang 2021	CGMCC 3.16003 T	China: Beijing	soil	MT077182	MT083924	MT083925	MT083926
*T. stellenboschensis* Visagie & K. Jacobs 2015	CBS 135665 T	South Africa	soil	JX091471	JX091605	JX140683	MN969157
*T. stipitatus* (Thom ex C.W. Emmons) C.R. Benj. 1955	CBS 375.48 T	USA	rotting wood	JN899348	KM111288	KF741957	KM023280
*T. stollii* N. Yilmaz et al. 2012	CBS 408.93 T	Netherlands	AIDS patient	JX315674	JX315633	JX315646	JX315712
*T. striatoconidius* Houbraken et al. 2020	CBS 550.89 T	Cuba	rotten leaves of *Pachyanthus poirettii*	MN431418	MN969441	MN969360	MT156347
*T. taiwanensis* K.W. Cheng & H.A. Ariyaw. 2025	NTUPPMCC 22-275 T	China: Taiwan	serpentine soil in rice field	PV476825	PV577091	PV550848	PV520157
*T. thailandensis* Manoch et al. 2013	CBS 133147 T	Thailand	forest soil	JX898041	JX494294	KF741940	KM023307
*T. tumuli* Jurjević & S.W. Peterson 2019	NRRL 62151 T	USA	soil from prairie	MH793071	MH792944	MH793008	MH793135
*T. veerkampii* N. Yilmaz et al. 2015	CBS 500.78 T	Colombia	cassava field soil	KF741984	KF741918	KF741961	KX961279
	**HLJ01-02**	China: Heilongjiang	soil at the riverside	**PP357623**	**PP373074**	**PP373079**	**PP373085**
*T. verruculosus* (Peyronel) Samson et al. 2011	NRRL 1050 T	USA	soil	KF741994	KF741928	KF741944	KM023306
*T. versatilis* Bridge & Buddie 2013	IMI 134755 T	UK	unknown	MN431395	MN969412	MN969319	MN969161
*T. virens* C. Liu et al. 2023	CGMCC 3.25207 T	China: Hainan	tidal flat sediments	ON563152	ON231297	ON470840	ON470841
*T. viridis* (Stolk & G.F. Orr) Arx 1987	CBS 114.72 T	Australia	soil	AF285782	JX494310	KF741935	JN121430
*T. viridulus* Samson et al. 2011	CBS 252.87 T	Australia	soil	JN899314	JX091385	KF741943	JF417422
*T. watsoniae* Y.P. Tan et al. 2024	MST FP2590 T	Australia	soil in a floodway	PP665736	PP682589	PP682561	PP682576
*T. wushanicus* X.C. Wang & W.Y. Zhuang 2021	CGMCC 3.20481 T	China: Chongqing	soil	MZ356356	MZ361347	MZ361354	MZ361361
*T. xishaensis* X.C. Wang et al. 2016	CGMCC 3.17995 T	China: Hainan	soil	KU644580	KU644581	KU644582	MZ361364
*T. yunnanensis* Doilom & C.F. Liao 2020	KUMCC 18-0208 T	China: Yunnan	rhizosphere soil of *Quercus rubra*	MT152339	MT161683	MT178251	n.a.
*T. zhenhaiensis* L. Wang 2022	CGMCC 3.16102 T	China: Zhejiang	mudflat soil	MZ045697	MZ054636	MZ054639	MZ054633
*T. helicus* (Raper & Fennel) C.R. Benj. 1955	CBS 335.48 T	Sweden	soil	JN899359	KJ865725	KJ885289	KM023273

GenBank accession numbers in bold indicate the newly generated sequences. “n.a.” is the abbreviation for “not available”.

**Table 7:** Detailed characteristics of the involved datasets.

Dataset	Gene Fragment	No. of Seq.	Length of Alignment (bp)	No. of Variable Sites	No. of Parsimony-Informative Sites	Model for ML	Model for BI
*Bacillispori*	*BenA*	10	457	170	97	TPM2 + G4	
	*CaM*	10	533	230	114	TN + G4	
	*RPB2*	10	1015	262	144	TN + G4	
	*BenA* + *CaM* + *RPB2*	10	2005	662	355	specified for the three loci	TrNef + I + G
*Helici* + *Brunneospori* + *Tenues*	*BenA*	16	521	243	181	TPM2u + G4	
	*CaM*	15	709	343	241	TN + I + G4	
	*RPB2*	16	973	317	256	HKY + I	
	*BenA* + *CaM* + *RPB2*	16	2203	903	678	specified	TrN + I + G
*Islandici*	*BenA*	41	468	233	184	HKY + I + G4	
	*CaM*	40	593	325	254	TN + I + G4	
	*RPB2*	40	1011	375	294	TN + I + G4	
	*BenA* + *CaM* + *RPB2*	41	2072	933	730	specified	TrNef + I + G
*Purpurei*	*BenA*	21	481	201	140	TPM2u + G4	
	*CaM*	18	564	274	198	TN + I	
	*RPB2*	21	1046	329	243	TN + G4	
	*BenA* + *CaM* + *RPB2*	21	2091	804	581	specified	GTR + I + G
*Subinflati*	*BenA*	12	383	133	74	K2P + I	
	*CaM*	12	533	232	150	TNe + G4	
	*RPB2*	12	1008	288	183	TN + I + G4	
	*BenA* + *CaM* + *RPB2*	12	1924	653	407	specified	TrNef + I + G
*Talaromyces*	*BenA*	117	550	271	201	TPM2u + I + G4	
	*CaM*	114	645	337	275	TIM3 + I + G4	
	*RPB2*	115	1050	395	343	TPM2u + I + G4	
	*BenA* + *CaM* + *RPB2*	117	2245	1003	819	specified	TVM + I + G

Abbreviations of models: GTR (General Time Reversible model), HKY (Hasegawa, Kishino and Yano model, i.e., unequal transition/transversion rates and unequal base frequency), K2P (Kimura 1980 model, i.e., unequal transition/transversion rates and equal base frequency), TIM (Transition model), TN/TrN (Tamura–Nei model), TNe/TrNef (equal-frequency Tamura–Nei model), TPM2 (AC=AT, AG=CT, CG=GT and equal base frequency), TPM2u (AC=AT, AG=CT, CG=GT and unequal base frequency), TVM (transversion model); +I (invariant sites); +G (gamma distribution).

**Table 8:** Morphological comparisons of new species and their closely related species.

Species	CYA 25 °C (mm)	CYA 37 °C (mm)	MEA (mm)	YES (mm)	Conidiophore	Conidia Shape	Conidia Wall	Conidia Size (µm)	Reference
***T. heilongjiangensis***	24–27	27–32	45–52	21–23	biverticillate or terverticillate	subglobose to ellipsoidal	rough	3.0–4.5 × 3.0–4.0	This study
*T. californicus*	25–40	30–40	40–51	n.a.	monoverticillate or biverticillate	globose to subglobose	finely rough to rough	4.0–6.0 × 4.0–7.0	[29]
*T. louisianensis*	35–39	37–43	45–55	n.a.	biverticillate	globose to subglobose	smooth to rough	3.5–5.0 × 3.5–5.0	[29]
*T. veerkampii*	20–31	18–23	38–42	35–46	biverticillate or monoverticillate	broadly ellipsoidal	finely rough	4.0–4.5 × 3.0–4.0	[30]
***T. tapisciae***	17–17	no growth	21–25	10–15	biverticillate, terverticillate or quaterverticillate	subglobose to ellipsoidal	rough	3.0–4.0 × 2.5–3.0	This study
*T. jiangxiensis*	n.a.	n.a.	26–33	n.a.	biverticillate	fusiform to pyriform, sometimes ellipsoidal	spiny	3.0–4.5 × 2.0–3.5	[31]

“n.a.” is the abbreviation for “not available”.

## Data Availability

The original data presented in the study are openly available in GenBank at https://www.ncbi.nlm.nih.gov (accessed on 15 April 2026).

## Supplementary Materials

The following supporting information can be downloaded at: https://www.mdpi.com/article/10.3390/jof12070485/s1, Figure S1. Maximum likelihood phylogeny of *Talaromyces* sect. *Bacillispori* inferred from *BenA* dataset. Bootstrap values ≥ 70% are indicated at nodes. Asterisk denotes 100% bootstrap; Figure S2. Maximum likelihood phylogeny of *Talaromyces* sect. *Bacillispori* inferred from *CaM* dataset. Bootstrap values ≥ 70% are indicated at nodes. Asterisk denotes 100% bootstrap; Figure S3. Maximum likelihood phylogeny of *Talaromyces* sect. *Bacillispori* inferred from *RPB2* dataset. Bootstrap values ≥ 70% are indicated at nodes. Asterisk denotes 100% bootstrap; Figure S4. Maximum likelihood phylogeny of sections *Brunneospori*, *Helici* and *Tenues* in *Talaromyces* inferred from *BenA* dataset. Bootstrap values ≥ 70% are indicated at nodes. Asterisk denotes 100% bootstrap; Figure S5. Maximum likelihood phylogeny of sections *Brunneospori*, *Helici* and *Tenues* in *Talaromyces* inferred from *CaM* dataset. Bootstrap values ≥ 70% are indicated at nodes. Asterisk denotes 100% bootstrap; Figure S6. Maximum likelihood phylogeny of sections *Brunneospori*, *Helici* and *Tenues* in *Talaromyces* inferred from *RPB2* dataset. Bootstrap values ≥ 70% are indicated at nodes. Asterisk denotes 100% bootstrap; Figure S7. Maximum likelihood phylogeny of *Talaromyces* sect. *Islandici* inferred from *BenA* dataset. Bootstrap values ≥ 70% are indicated at nodes. Asterisk denotes 100% bootstrap; Figure S8. Maximum likelihood phylogeny of *Talaromyces* sect. *Islandici* inferred from *CaM* dataset. Bootstrap values ≥ 70% are indicated at nodes. Asterisk denotes 100% bootstrap; Figure S9. Maximum likelihood phylogeny of *Talaromyces* sect. *Islandici* inferred from *RPB2* dataset. Bootstrap values ≥ 70% are indicated at nodes. Asterisk denotes 100% bootstrap; Figure S10. Maximum likelihood phylogeny of *Talaromyces* sect. *Purpurei* inferred from *BenA* dataset. Bootstrap values ≥ 70% are indicated at nodes. Asterisk denotes 100% bootstrap; Figure S11. Maximum likelihood phylogeny of *Talaromyces* sect. *Purpurei* inferred from *CaM* dataset. Bootstrap values ≥ 70% are indicated at nodes. Asterisk denotes 100% bootstrap; Figure S12. Maximum likelihood phylogeny of *Talaromyces* sect. *Purpurei* inferred from *RPB2* dataset. Bootstrap values ≥ 70% are indicated at nodes. Asterisk denotes 100% bootstrap; Figure S13. Maximum likelihood phylogeny of *Talaromyces* sect. *Subinflati* inferred from *BenA* dataset. Bootstrap values ≥ 70% are indicated at nodes. Asterisk denotes 100% bootstrap; Figure S14. Maximum likelihood phylogeny of *Talaromyces* sect. *Subinflati* inferred from *CaM* dataset. Bootstrap values ≥ 70% are indicated at nodes. Asterisk denotes 100% bootstrap; Figure S15. Maximum likelihood phylogeny of *Talaromyces* sect. *Subinflati* inferred from *RPB2* dataset. Bootstrap values ≥ 70% are indicated at nodes. Asterisk denotes 100% bootstrap; Figure S16. Maximum likelihood phylogeny of *Talaromyces* sect. *Talaromyces* inferred from *BenA* dataset. Bootstrap values ≥ 70% are indicated at nodes. Asterisk denotes 100% bootstrap; Figure S17. Maximum likelihood phylogeny of *Talaromyces* sect. *Talaromyces* inferred from *CaM* dataset. Bootstrap values ≥ 70% are indicated at nodes. Asterisk denotes 100% bootstrap; Figure S18. Maximum likelihood phylogeny of *Talaromyces* sect. *Talaromyces* inferred from *RPB2* dataset. Bootstrap values ≥ 70% are indicated at nodes. Asterisk denotes 100% bootstrap.
