# Supplementary material for: An Updated Taxonomy of Talaromyces (Trichocomaceae, Eurotiales): New Series and Species
Source: J Fungi (Basel). 2026 Jul 1;12(7):485. doi: 10.3390/jof12070485 (PMC13413039; doi:10.3390/jof12070485)
Supplement: Supplementary file 1 [file jof-12-00485-s001.zip › Supplementary legends.pdf]

Figure S1. Maximum likelihood phylogeny of *Talaromyces* sect. *Bacillispori* inferred from BenA dataset. Bootstrap values ≥ 70% are indicated at nodes. Asterisk denotes 100% bootstrap.

Figure S2. Maximum likelihood phylogeny of *Talaromyces* sect. *Bacillispori* inferred from CaM dataset. Bootstrap values ≥ 70% are indicated at nodes. Asterisk denotes 100% bootstrap.

Figure S3. Maximum likelihood phylogeny of *Talaromyces* sect. *Bacillispori* inferred from RPB2 dataset. Bootstrap values ≥ 70% are indicated at nodes. Asterisk denotes 100% bootstrap.

Figure S4. Maximum likelihood phylogeny of sections *Brunneospori*, *Helici* and *Tenues* in *Talaromyces* inferred from BenA dataset. Bootstrap values ≥ 70% are indicated at nodes. Asterisk denotes 100% bootstrap.

Figure S5. Maximum likelihood phylogeny of sections *Brunneospori*, *Helici* and *Tenues* in *Talaromyces* inferred from CaM dataset. Bootstrap values ≥ 70% are indicated at nodes. Asterisk denotes 100% bootstrap.

Figure S6. Maximum likelihood phylogeny of sections *Brunneospori*, *Helici* and *Tenues* in *Talaromyces* inferred from RPB2 dataset. Bootstrap values ≥ 70% are indicated at nodes. Asterisk denotes 100% bootstrap.

Figure S7. Maximum likelihood phylogeny of *Talaromyces* sect. *Islandici* inferred from BenA dataset. Bootstrap values ≥ 70% are indicated at nodes. Asterisk denotes 100% bootstrap.

Figure S8. Maximum likelihood phylogeny of *Talaromyces* sect. *Islandici* inferred from CaM dataset. Bootstrap values ≥ 70% are indicated at nodes. Asterisk denotes 100% bootstrap.

Figure S9. Maximum likelihood phylogeny of *Talaromyces* sect. *Islandici* inferred from RPB2 dataset. Bootstrap values ≥ 70% are indicated at nodes. Asterisk denotes 100% bootstrap.

Figure S10. Maximum likelihood phylogeny of *Talaromyces* sect. *Purpurei* inferred from BenA dataset. Bootstrap values ≥ 70% are indicated at nodes. Asterisk denotes 100% bootstrap.

Figure S11. Maximum likelihood phylogeny of *Talaromyces* sect. *Purpurei* inferred from CaM dataset. Bootstrap values ≥ 70% are indicated at nodes. Asterisk denotes 100% bootstrap.

Figure S12. Maximum likelihood phylogeny of *Talaromyces* sect. *Purpurei* inferred from RPB2 dataset. Bootstrap values ≥ 70% are indicated at nodes. Asterisk denotes 100% bootstrap.

Figure S13. Maximum likelihood phylogeny of *Talaromyces* sect. *Subinflati* inferred from BenA dataset. Bootstrap values ≥ 70% are indicated at nodes. Asterisk denotes 100% bootstrap.

Figure S14. Maximum likelihood phylogeny of *Talaromyces* sect. *Subinflati* inferred from CaM dataset. Bootstrap values ≥ 70% are indicated at nodes. Asterisk denotes 100% bootstrap.

Figure S15. Maximum likelihood phylogeny of *Talaromyces* sect. *Subinflati* inferred from RPB2 dataset. Bootstrap values ≥ 70% are indicated at nodes. Asterisk denotes 100% bootstrap.

Figure S16. Maximum likelihood phylogeny of *Talaromyces* sect. *Talaromyces* inferred from BenA dataset. Bootstrap values ≥ 70% are indicated at nodes. Asterisk denotes 100% bootstrap.

Figure S17. Maximum likelihood phylogeny of *Talaromyces* sect. *Talaromyces* inferred from CaM dataset. Bootstrap values ≥ 70% are indicated at nodes. Asterisk denotes 100% bootstrap.

Figure S18. Maximum likelihood phylogeny of *Talaromyces* sect. *Talaromyces* inferred from RPB2 dataset. Bootstrap values ≥ 70% are indicated at nodes. Asterisk denotes 100% bootstrap.
