# Supplementary figures and images for: An Updated Taxonomy of Talaromyces (Trichocomaceae, Eurotiales): New Series and Species
Source: J Fungi (Basel). 2026 Jul 1;12(7):485. doi: 10.3390/jof12070485 (PMC13413039; doi:10.3390/jof12070485)

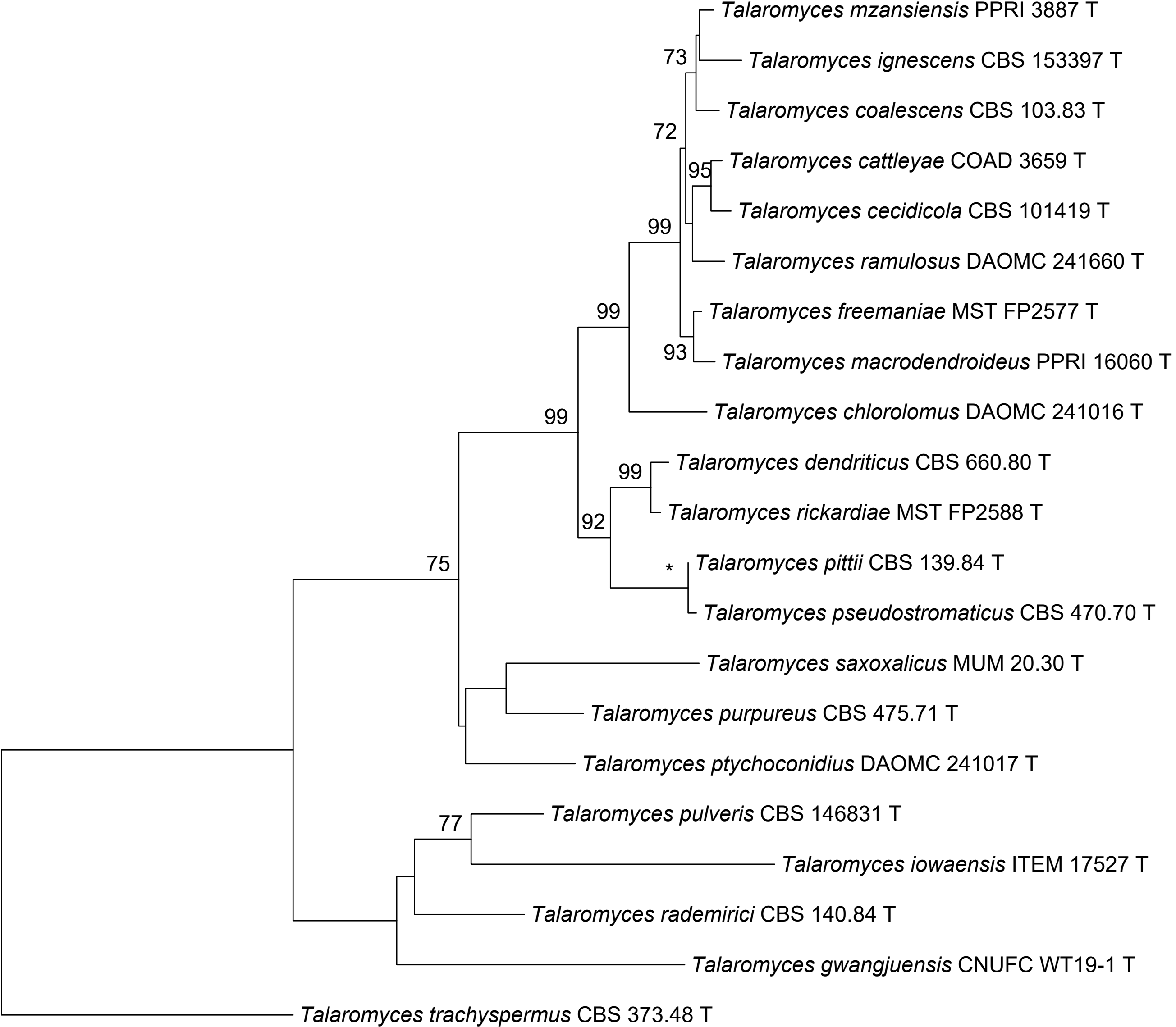

0.06

Supplement: Supplementary file 1 [file jof-12-00485-s001.zip › FigureS10_Purpurei_BenA.pdf]

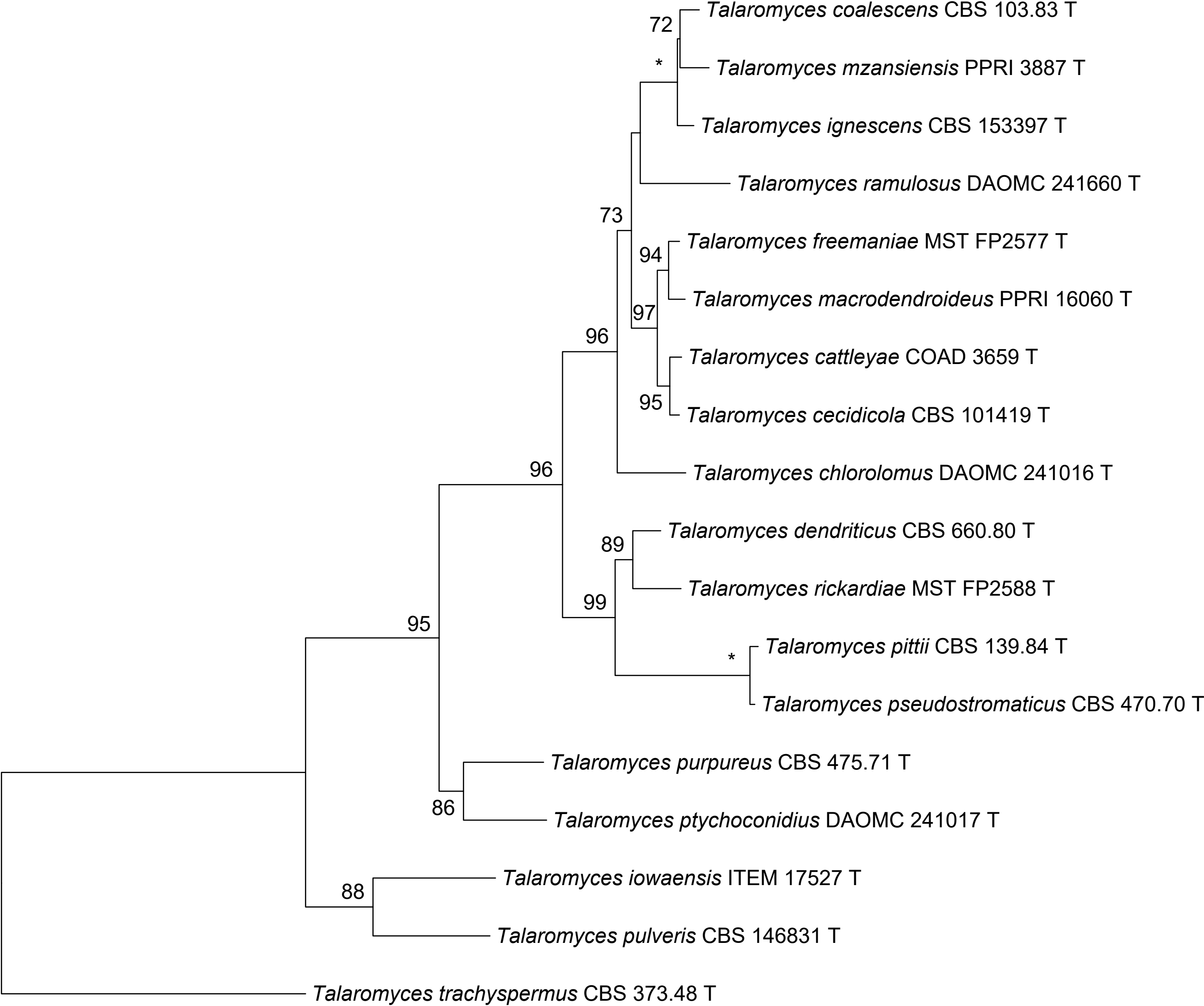

0.07

Supplement: Supplementary file 1 [file jof-12-00485-s001.zip › FigureS11_Purpurei_CaM.pdf]

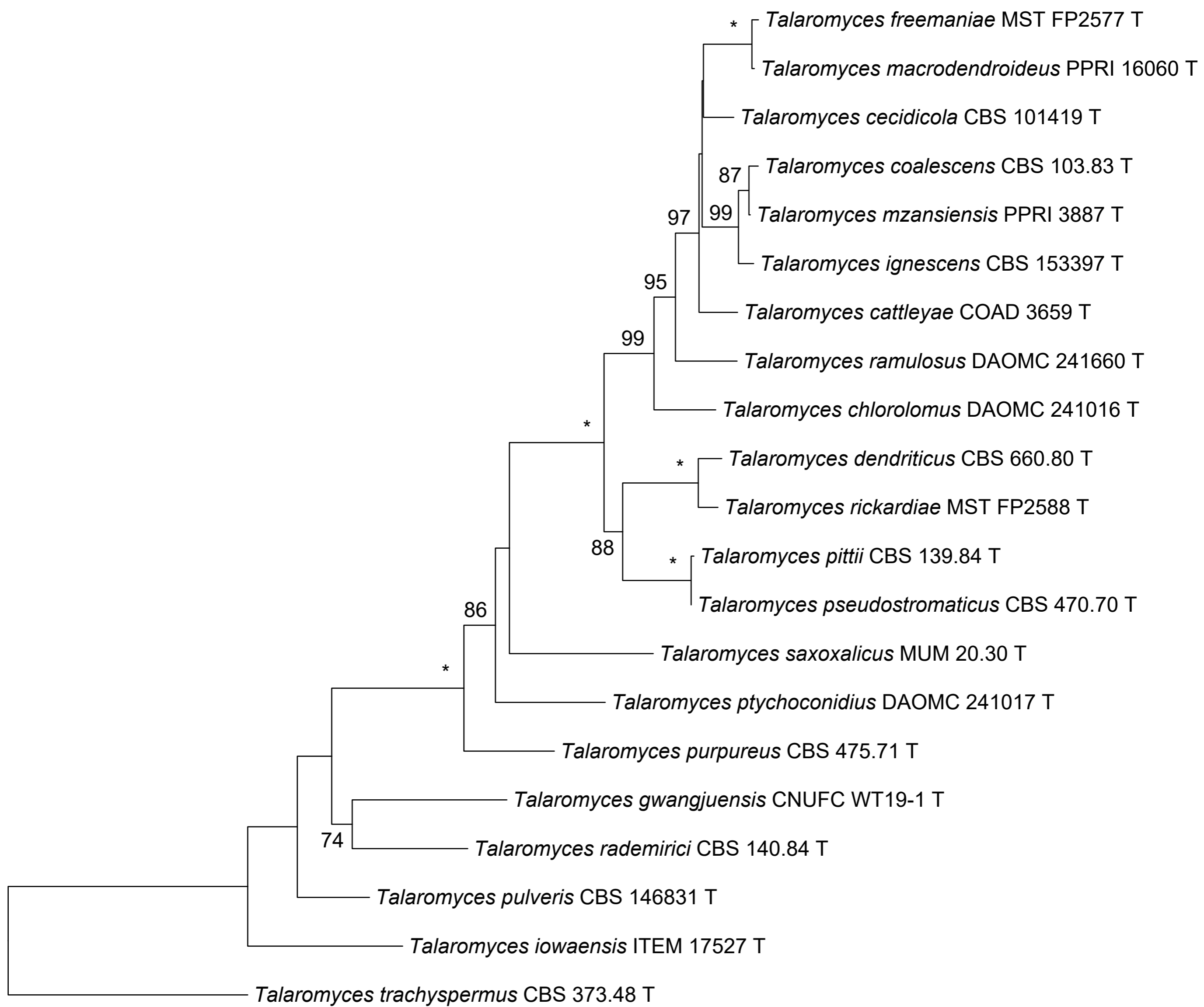

0.04

Supplement: Supplementary file 1 [file jof-12-00485-s001.zip › FigureS12_Purpurei_RPB2.pdf]

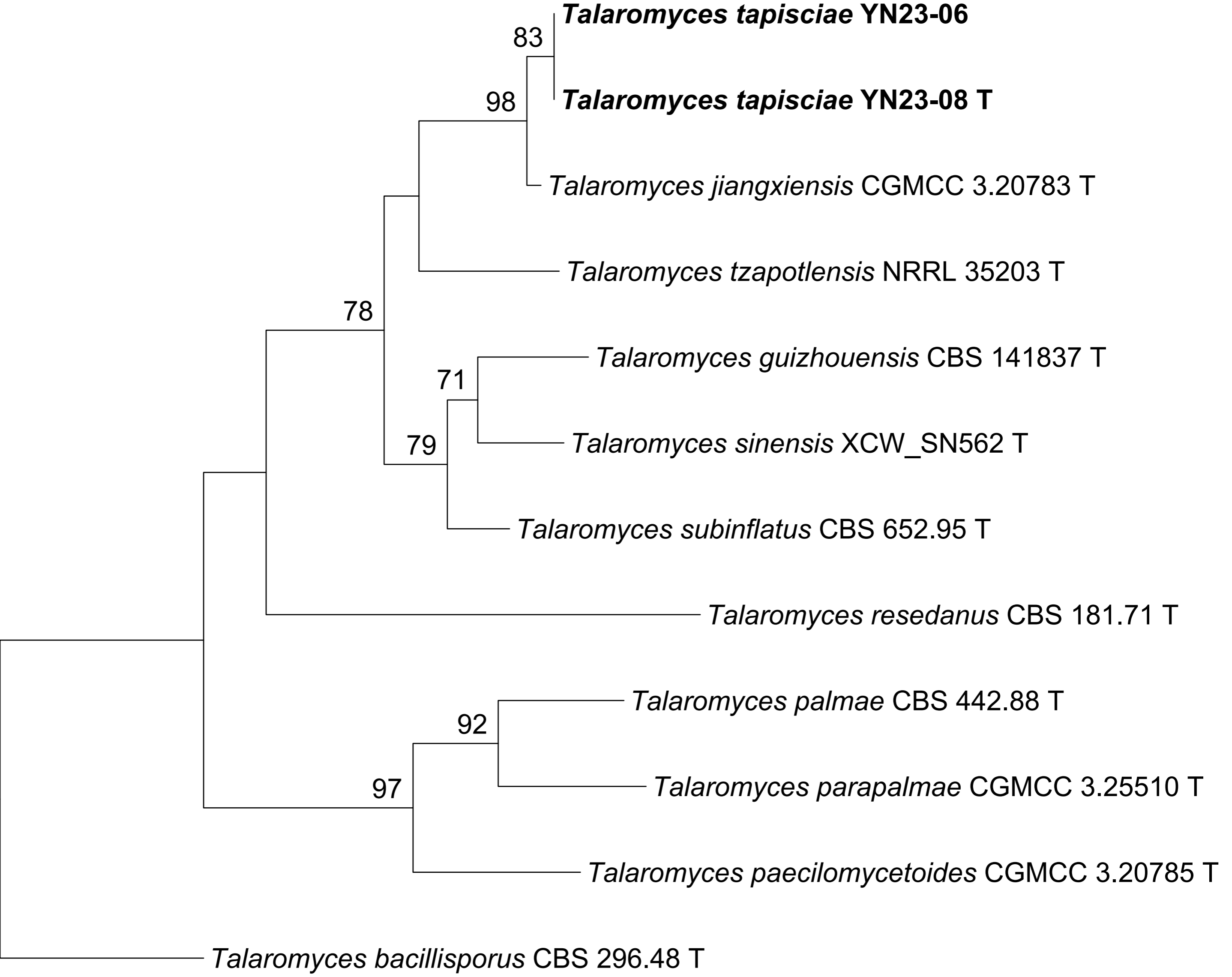

0.03

Supplement: Supplementary file 1 [file jof-12-00485-s001.zip › FigureS13_Subinflati_BenA.pdf]

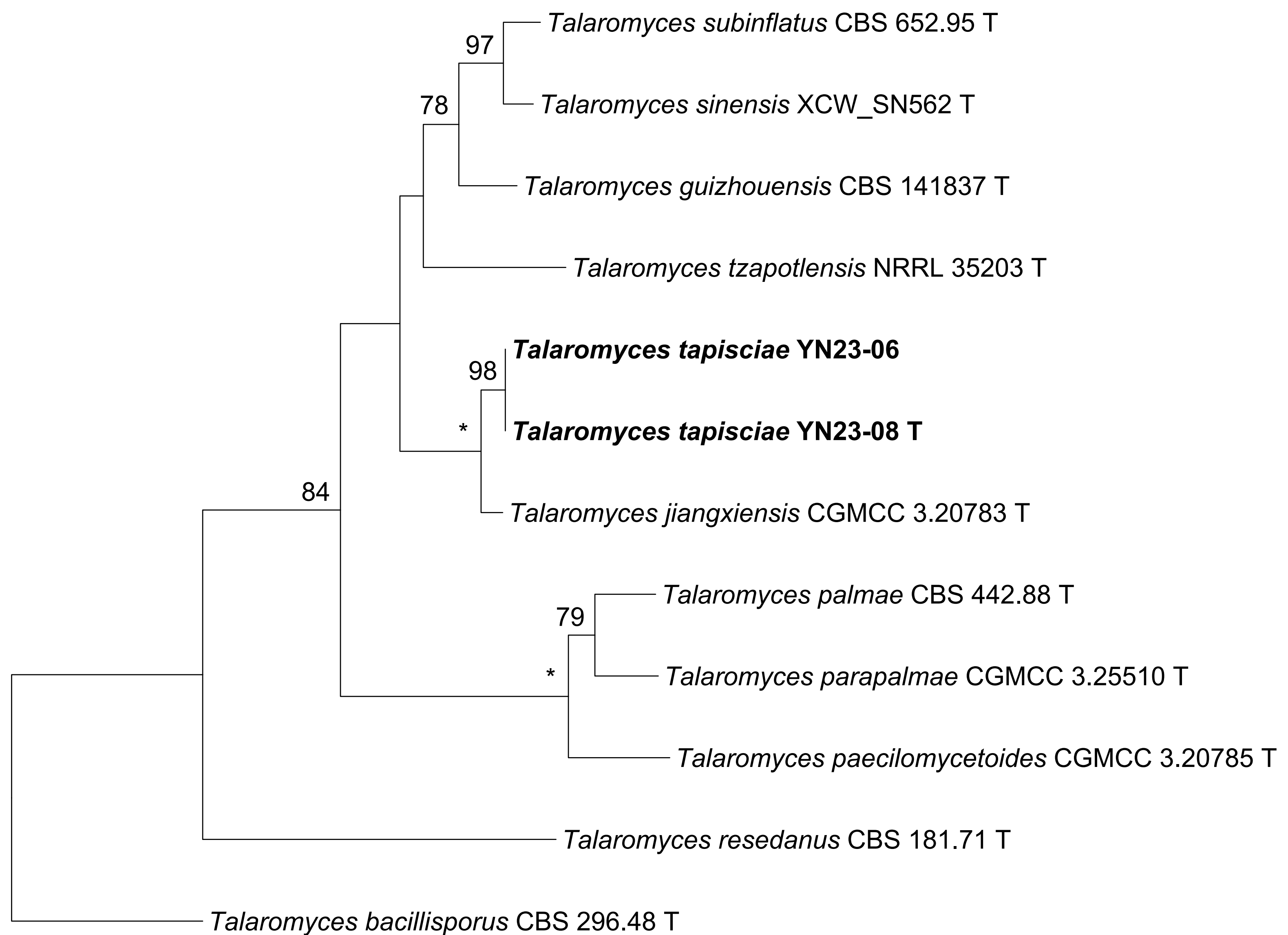

Supplement: Supplementary file 1 [file jof-12-00485-s001.zip › FigureS14_Subinflati_CaM.pdf]

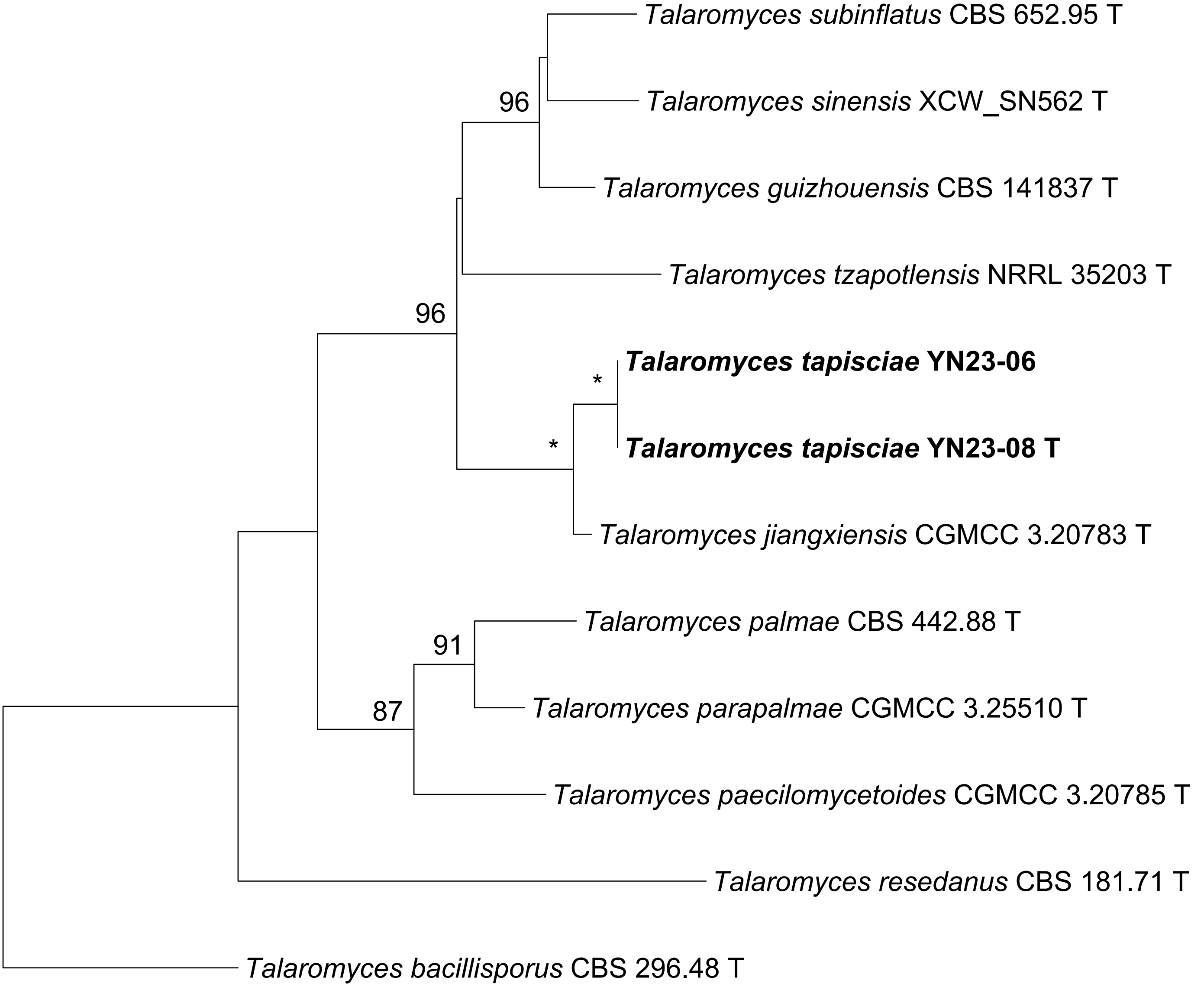

0.03

Supplement: Supplementary file 1 [file jof-12-00485-s001.zip › FigureS15_Subinflati_RPB2.pdf]

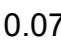

Supplement: Supplementary file 1 [file jof-12-00485-s001.zip › FigureS16_Talaromyces_BenA.pdf]

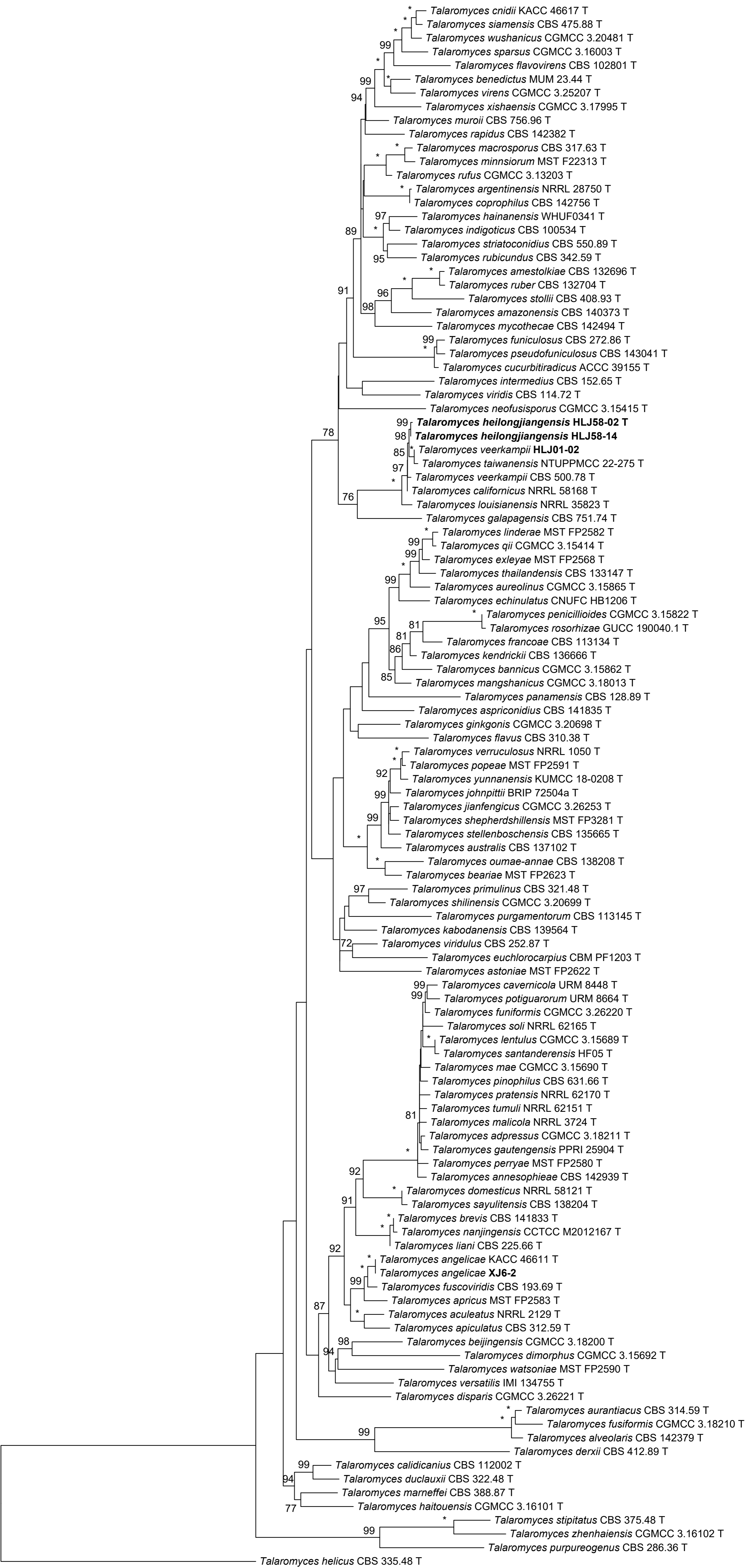

Supplement: Supplementary file 1 [file jof-12-00485-s001.zip › FigureS17_Talaromyces_CaM.pdf]

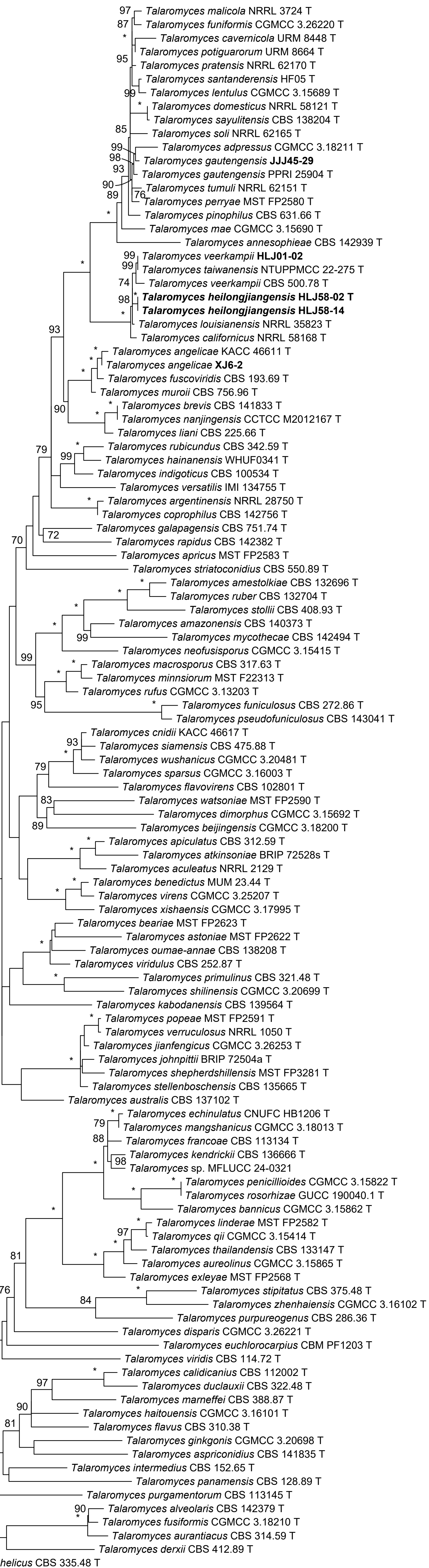

0.04

Supplement: Supplementary file 1 [file jof-12-00485-s001.zip › FigureS18_Talaromyces_RPB2.pdf]

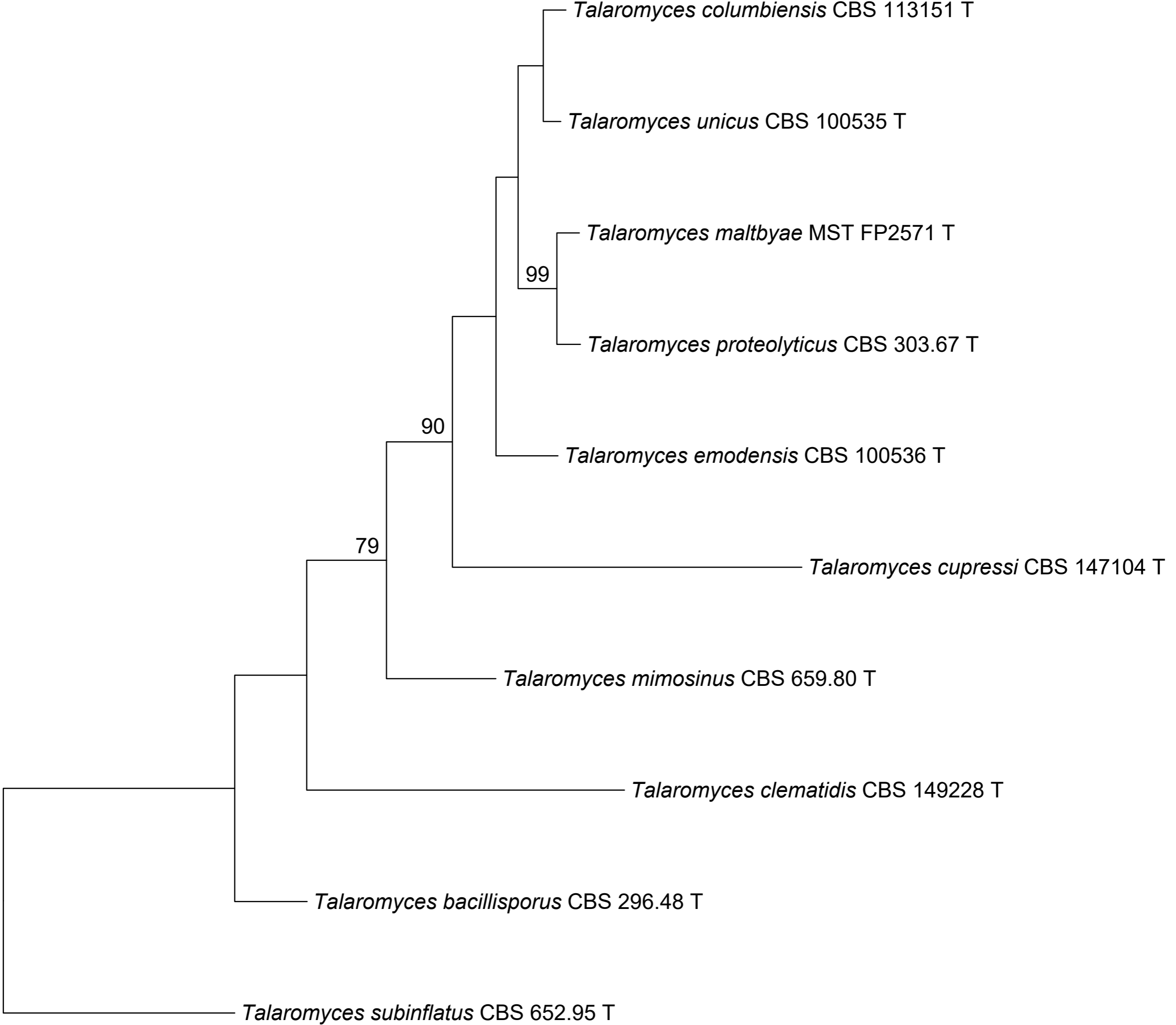

0.04

Supplement: Supplementary file 1 [file jof-12-00485-s001.zip › FigureS1_Bacillispori_BenA.pdf]

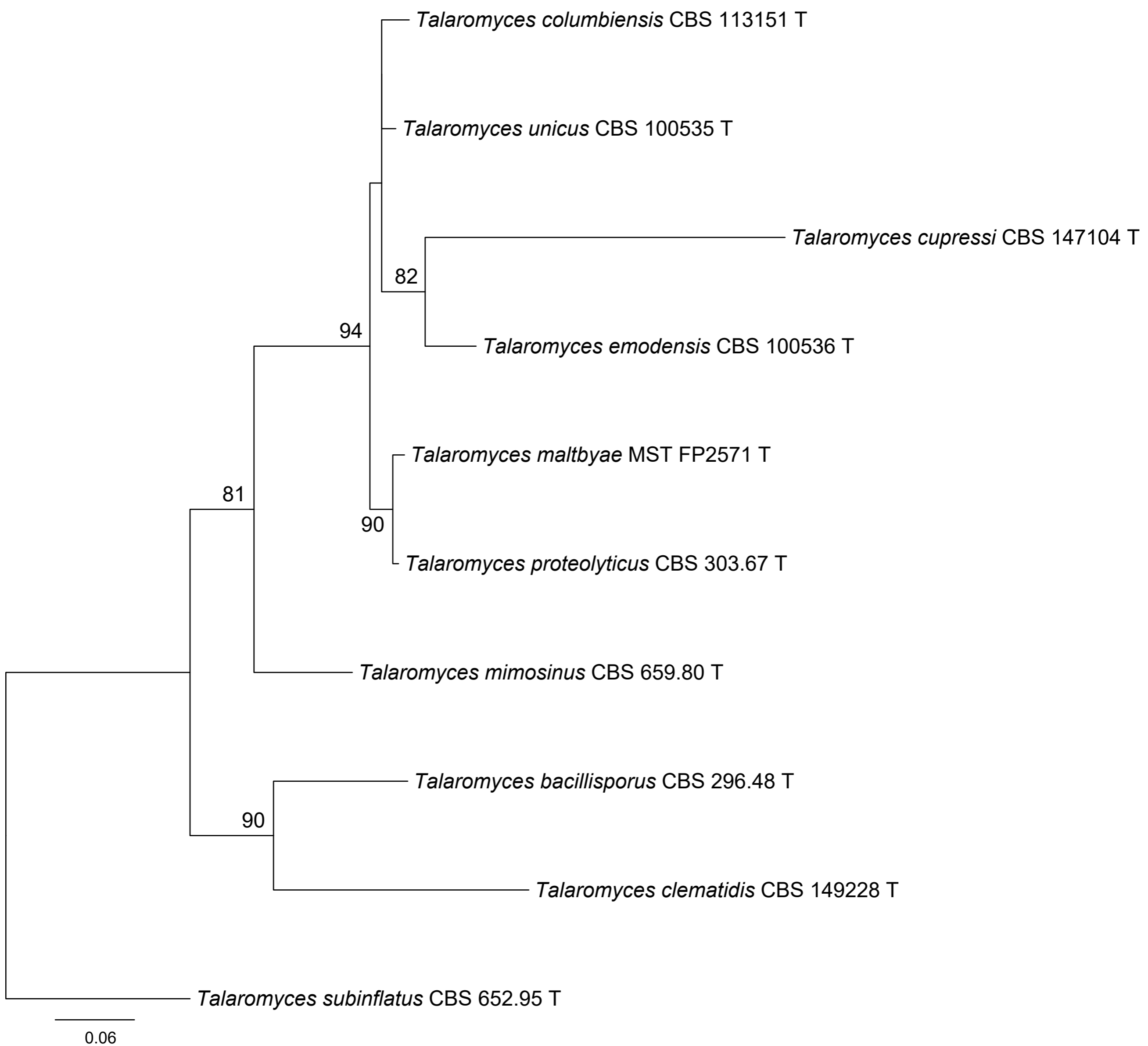

Supplement: Supplementary file 1 [file jof-12-00485-s001.zip › FigureS2_Bacillispori_CaM.pdf]

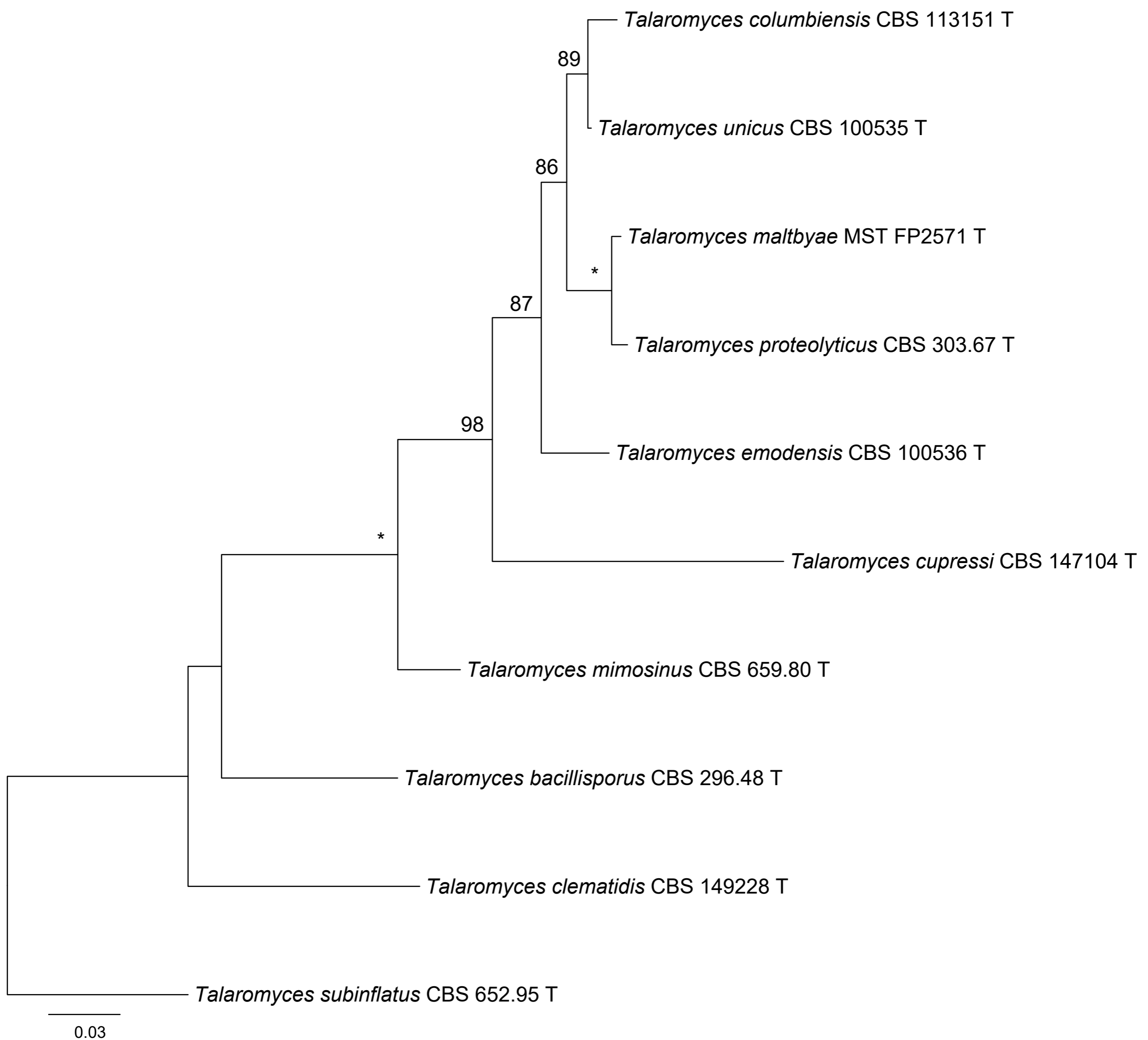

Supplement: Supplementary file 1 [file jof-12-00485-s001.zip › FigureS3_Bacillispori_RPB2.pdf]

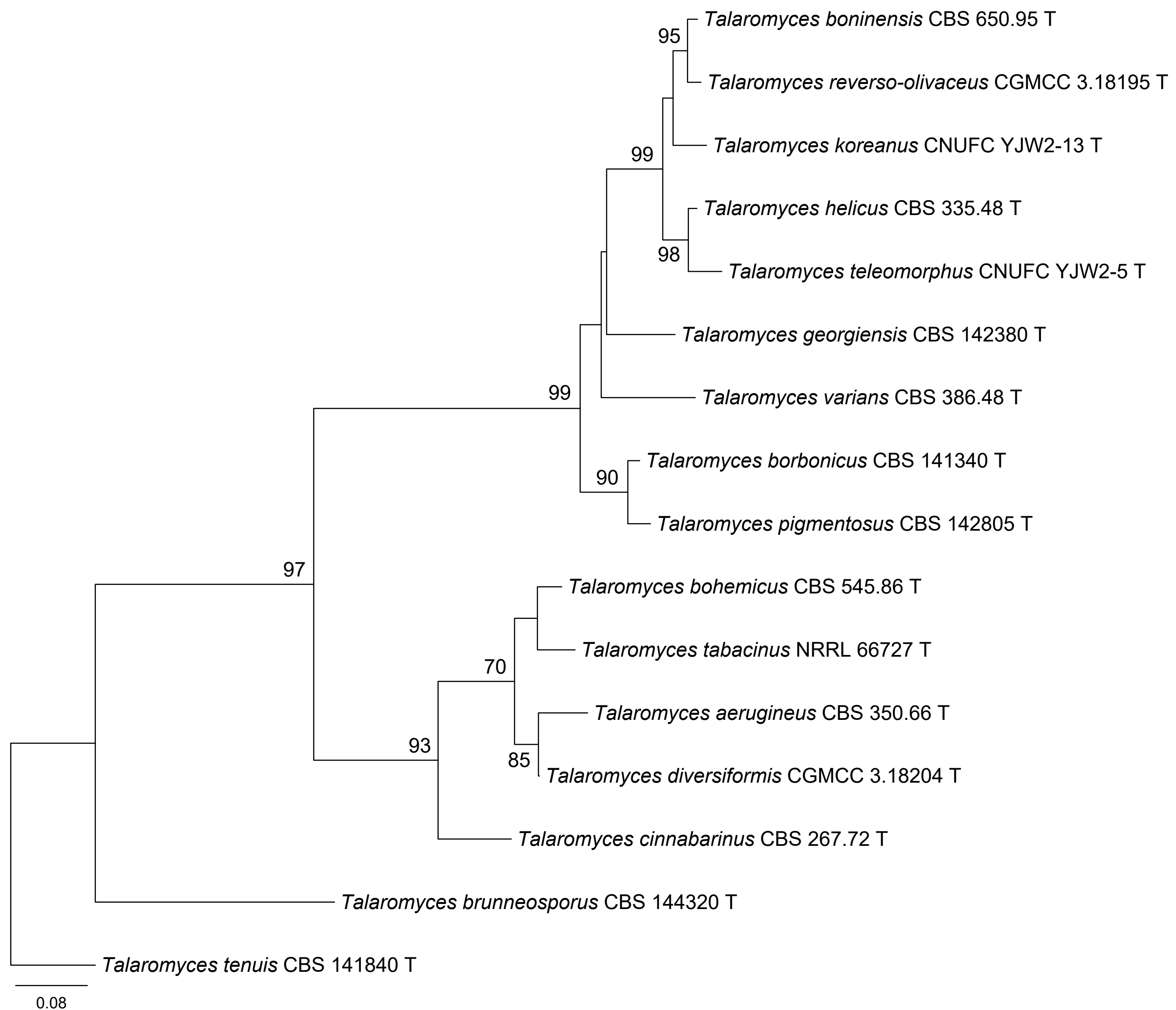

Supplement: Supplementary file 1 [file jof-12-00485-s001.zip › FigureS4_Helici_BenA.pdf]

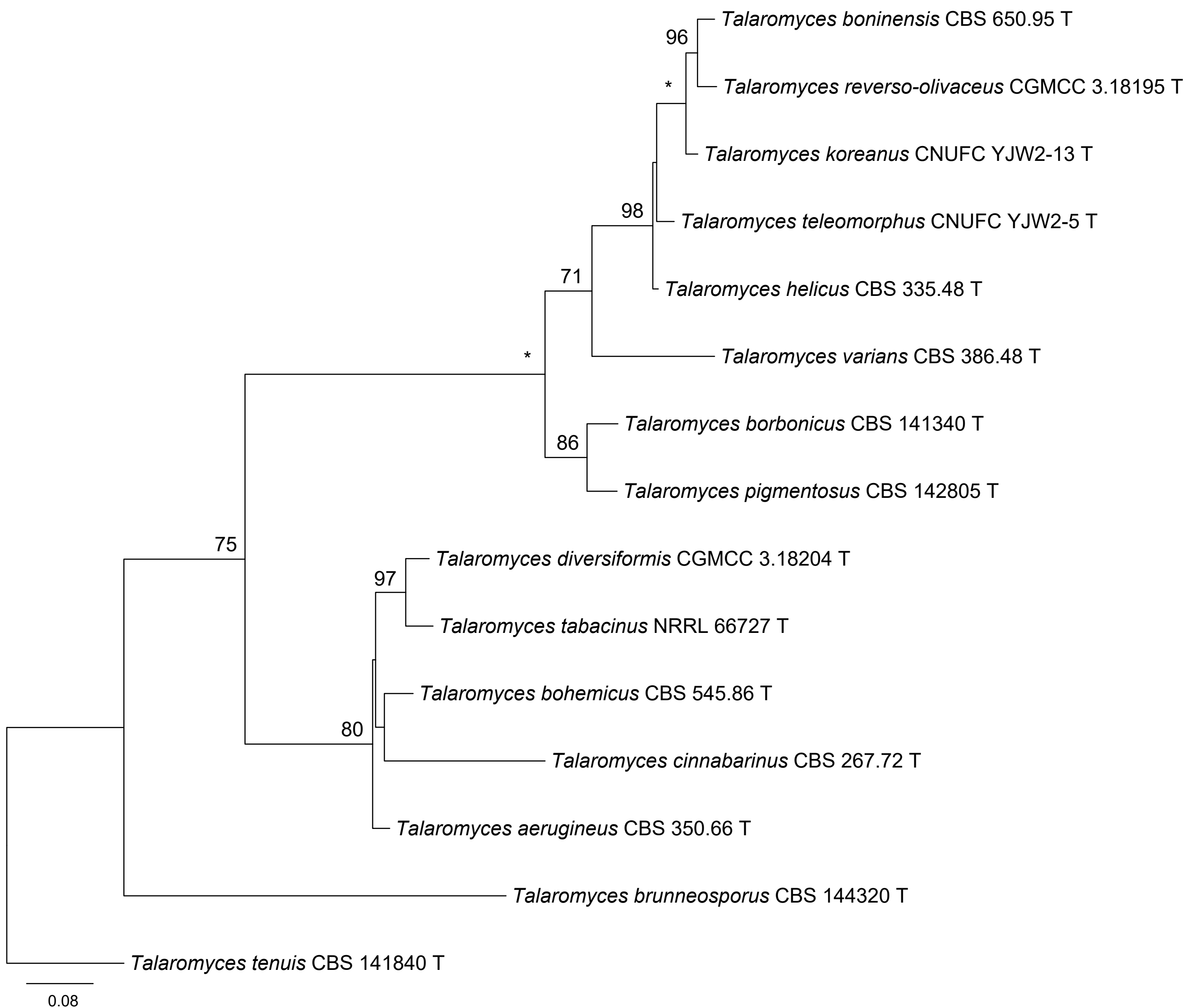

Supplement: Supplementary file 1 [file jof-12-00485-s001.zip › FigureS5_Helici_CaM.pdf]

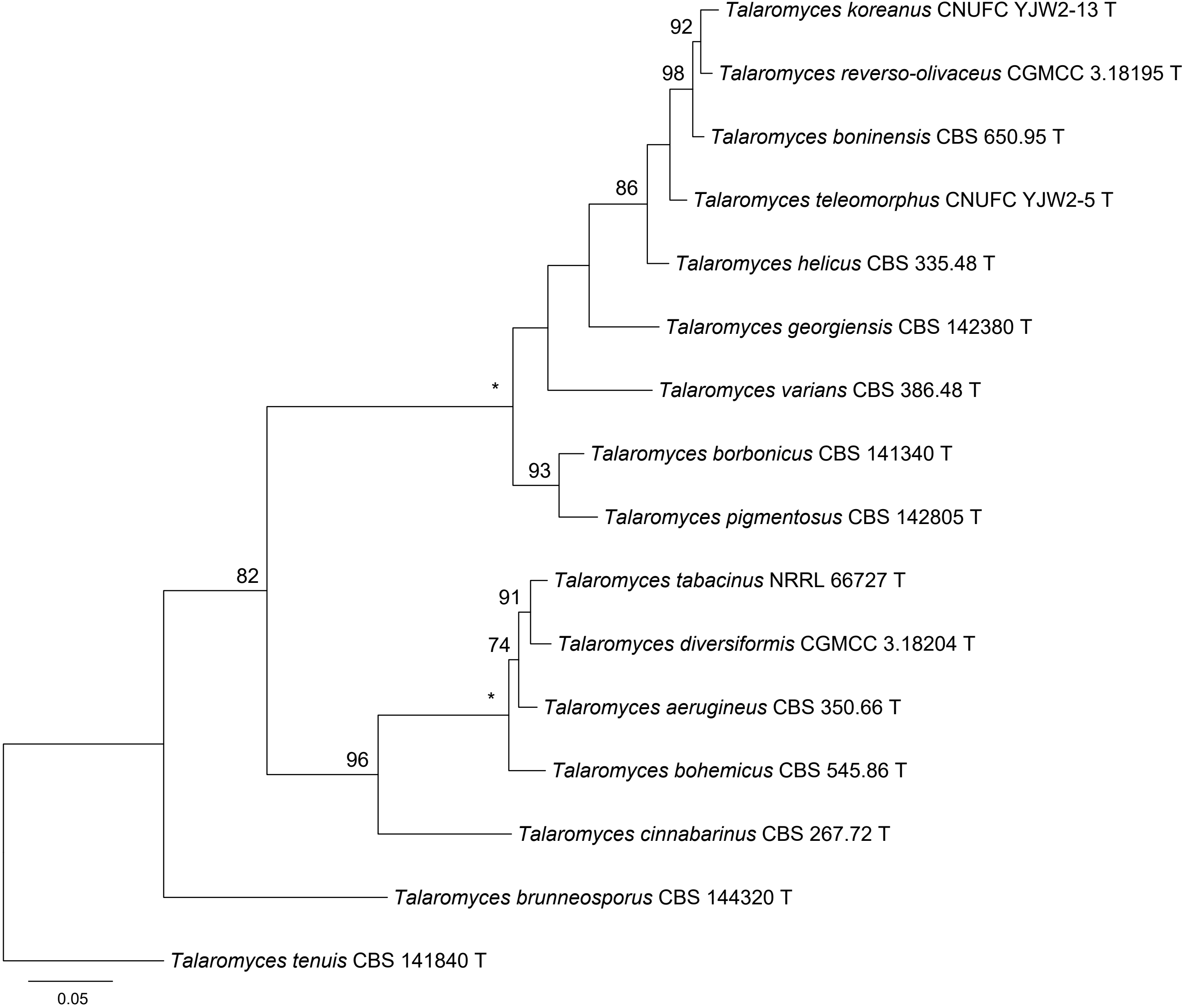

Supplement: Supplementary file 1 [file jof-12-00485-s001.zip › FigureS6_Helici_RPB2.pdf]

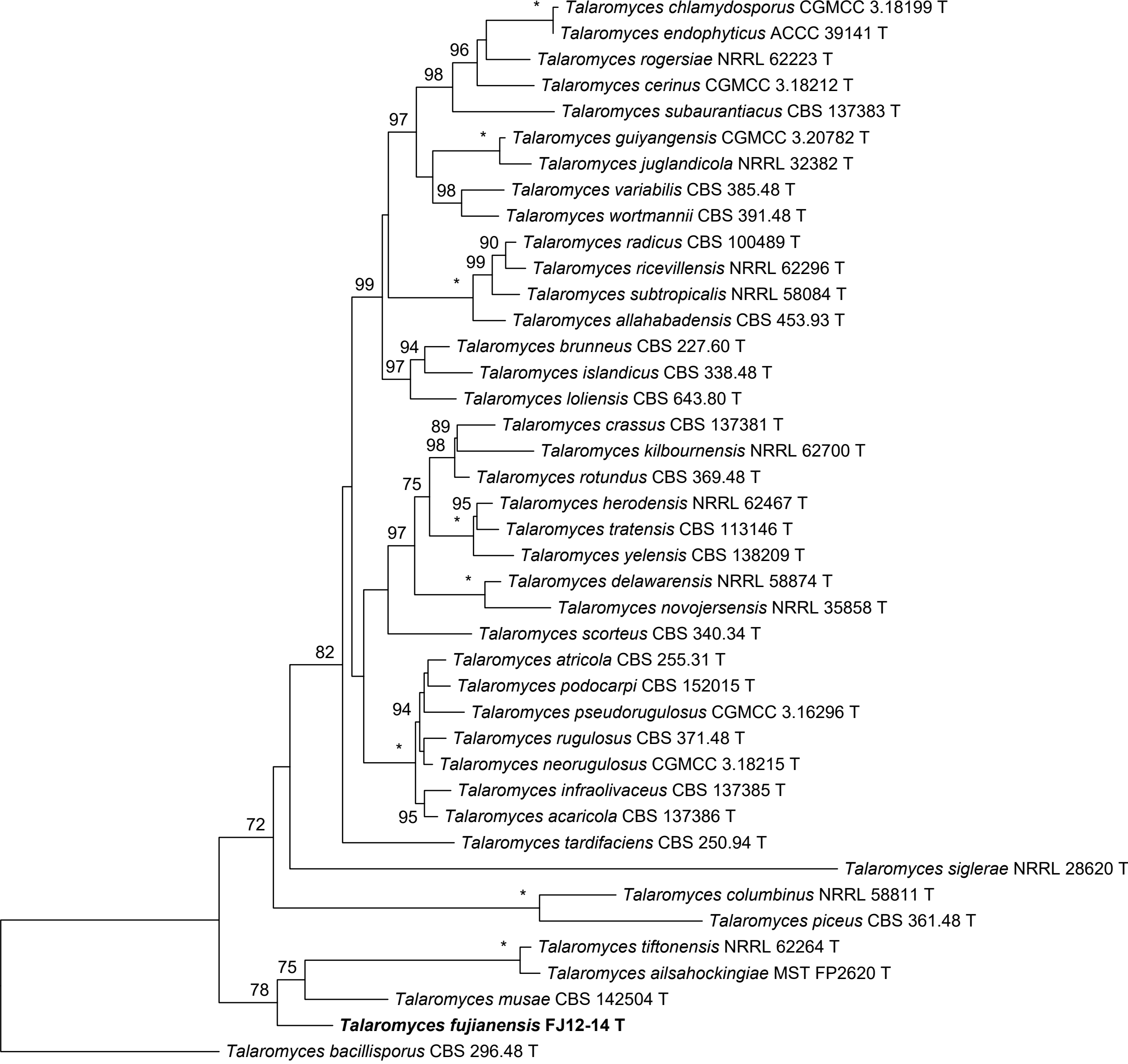

0.05

Supplement: Supplementary file 1 [file jof-12-00485-s001.zip › FigureS7_Islandici_BenA.pdf]

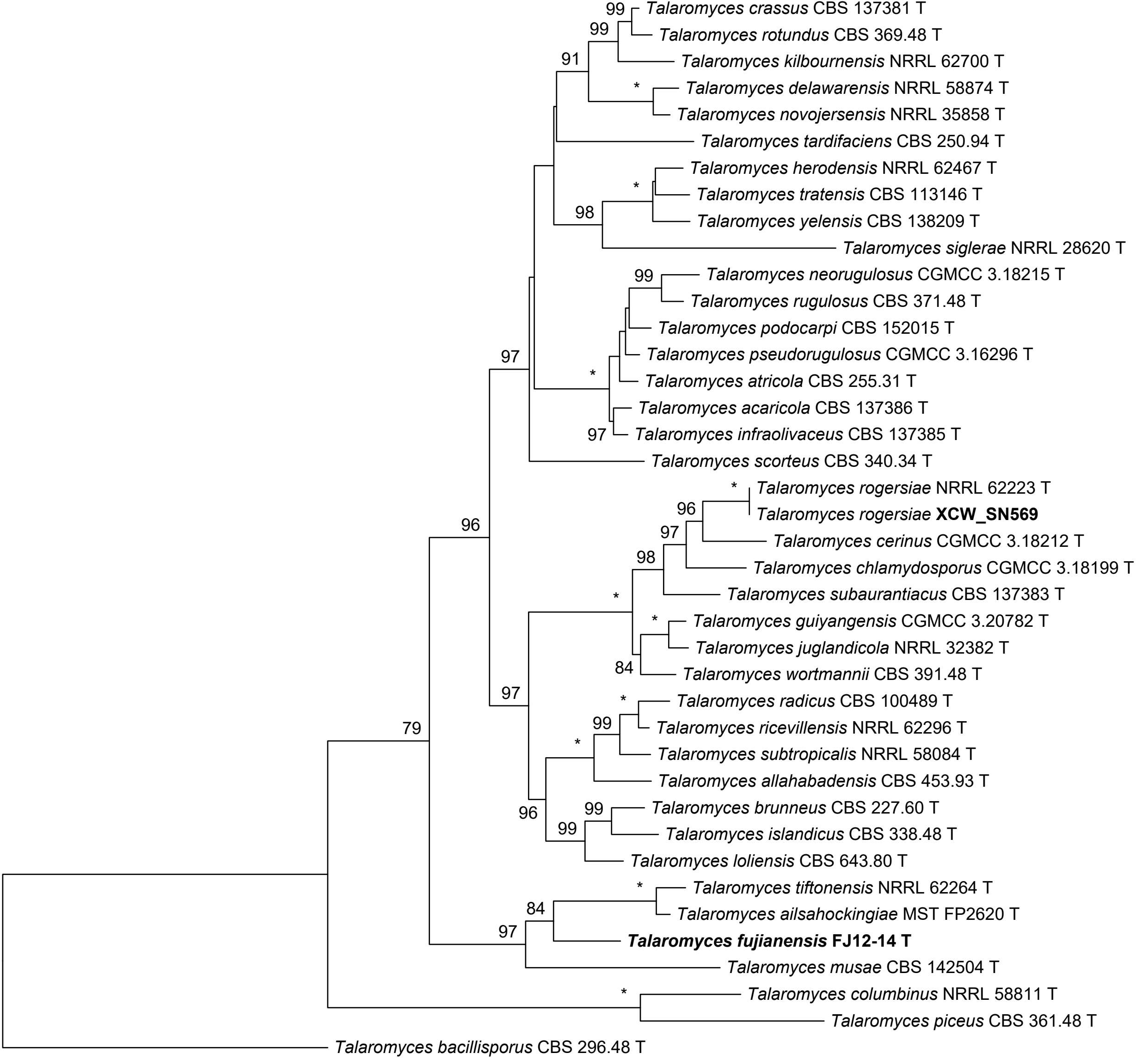

0.04

Supplement: Supplementary file 1 [file jof-12-00485-s001.zip › FigureS9_Islandici_RPB2.pdf]
