# Supplementary material for: An Updated Taxonomy of Talaromyces (Trichocomaceae, Eurotiales): New Series and Species
Source: J Fungi (Basel). 2026 Jul 1;12(7):485. doi: 10.3390/jof12070485 (PMC13413039; doi:10.3390/jof12070485)
Supplement: Supplementary file 1 [file FigureS8_Islandici_CaM.pdf › FigureS8_Islandici_CaM.pdf]

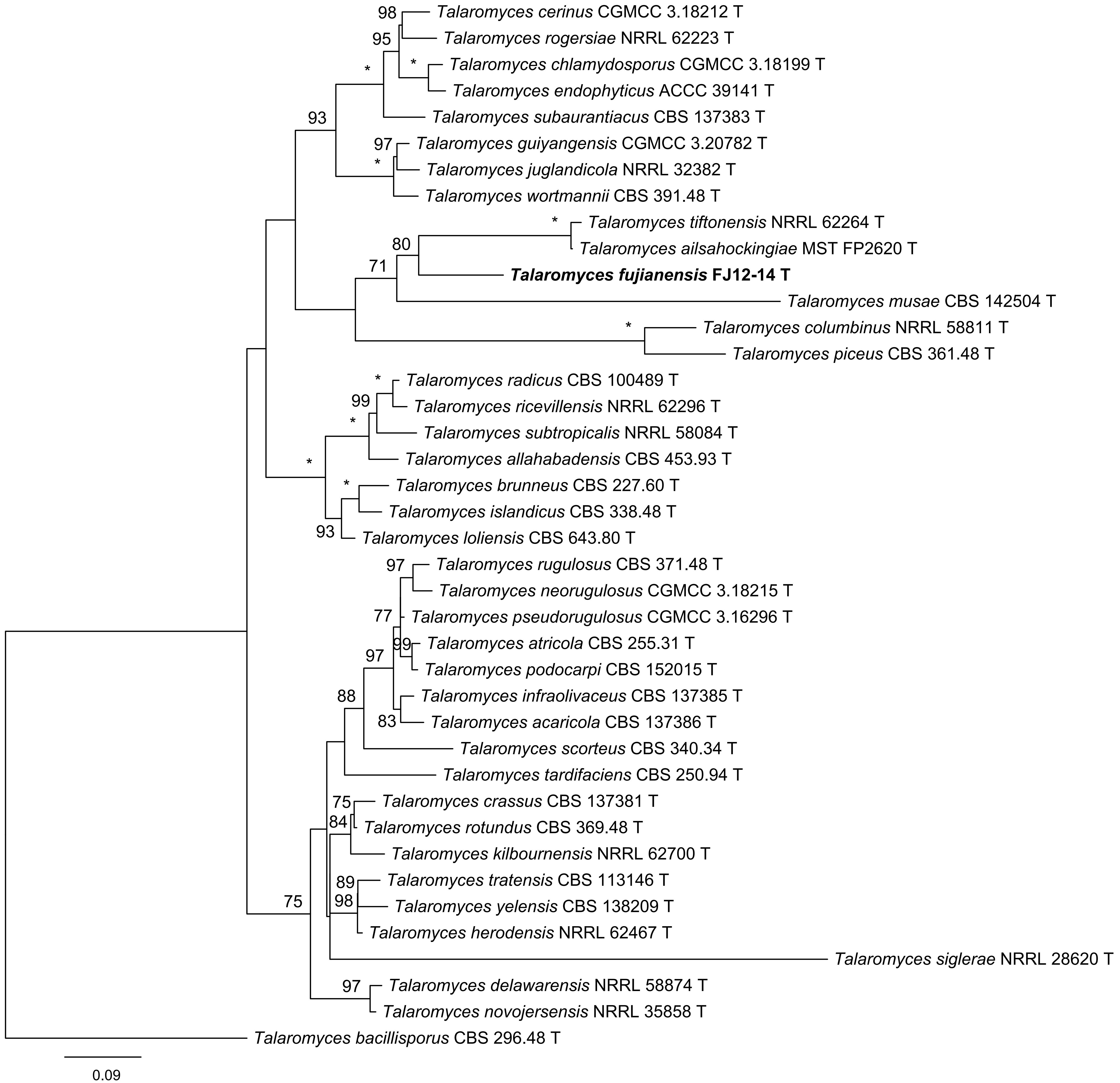

Talaromyces cerinus CGMCC 3.18212 T
Talaromyces rogersiae NRRL 62223 T
Talaromyces chlamydosporus CGMCC 3.18199 T
Talaromyces endophyticus ACCC 39141 T
Talaromyces subaurantiacus CBS 137383 T
Talaromyces guiyangensis CGMCC 3.20782 T
Talaromyces juglandicola NRRL 32382 T
Talaromyces wortmannii CBS 391.48 T
Talaromyces tiftonensis NRRL 62264 T
Talaromyces ailsahockingiae MST FP2620 T
Talaromyces fujianensis FJ12-14 T
Talaromyces musae CBS 142504 T
Talaromyces columbinus NRRL 58811 T
Talaromyces piceus CBS 361.48 T
Talaromyces radicus CBS 100489 T
Talaromyces ricevillensis NRRL 62296 T
Talaromyces subtropicalis NRRL 58084 T
Talaromyces allahabadensis CBS 453.93 T
Talaromyces brunneus CBS 227.60 T
Talaromyces islandicus CBS 338.48 T
Talaromyces loliensis CBS 643.80 T
Talaromyces rugulosus CBS 371.48 T
Talaromyces neorugulosus CGMCC 3.18215 T
Talaromyces pseudorugulosus CGMCC 3.16296 T
Talaromyces atricola CBS 255.31 T
Talaromyces podocarpi CBS 152015 T
Talaromyces infraolivaceus CBS 137385 T
Talaromyces acaricola CBS 137386 T
Talaromyces scorteus CBS 340.34 T
Talaromyces tardifaciens CBS 250.94 T
Talaromyces crassus CBS 137381 T
Talaromyces rotundus CBS 369.48 T
Talaromyces kilbournensis NRRL 62700 T
Talaromyces tratensis CBS 113146 T
Talaromyces yelensis CBS 138209 T
Talaromyces herodensis NRRL 62467 T
Talaromyces siglerae NRRL 28620 T
Talaromyces delawarensis NRRL 58874 T
Talaromyces novojersensis NRRL 35858 T
Talaromyces bacillisporus CBS 296.48 T
98
95
*
*
93
97
*
*
80
71
*
*
99
*
*
*
93
97
77
99
97
83
88
75
84
89
98
75
97
0.09
